# Renal fibroblasts are involved in fibrogenic changes in kidney fibrosis associated with dysfunctional telomeres

**DOI:** 10.1038/s12276-024-01318-8

**Published:** 2024-10-01

**Authors:** Sarita Saraswati, Paula Martínez, Rosa Serrano, Diego Mejías, Osvaldo Graña-Castro, Ruth Álvarez Díaz, Maria A. Blasco

**Affiliations:** 1 Telomeres and Telomerase Group–Fundacion Humanismo y Ciencia, Molecular Oncology Program, Spanish National Cancer Centre (CNIO), Melchor Fernández Almagro 3, Madrid, E-28029 Spain; 2 Confocal Microscopy Unit, Biotechnology Program, Spanish National Cancer Centre (CNIO), Melchor Fernández Almagro 3, Madrid, E-28029 Spain; 3 Advanced Optical Microscopy Unit, UCCTs, Instituto de Salud Carlos III (ISCIII), E-28220 Majadahonda, Madrid Spain; 4 Bioinformatics Unit, Structural Biology and Biocomputing Program, Spanish National Cancer Centre (CNIO), Melchor Fernández Almagro 3, Madrid, E-28029 Spain; 5 Department of Basic Medical Sciences, Institute of Applied Molecular Medicine (IMMA-Nemesio Díez), School of Medicine, San Pablo-CEU University, CEU Universities, Boadilla del Monte, Madrid, Spain

**Keywords:** Mechanisms of disease, Gene expression

## Abstract

Tubulointerstitial fibrosis associated with chronic kidney disease (CKD) represents a global health care problem. We previously reported that short and dysfunctional telomeres lead to interstitial renal fibrosis; however, the cell-of-origin of kidney fibrosis associated with telomere dysfunction is currently unknown. We induced telomere dysfunction by deleting the *Trf1* gene encoding a telomere-binding factor specifically in renal fibroblasts in both short-term and long-term life-long experiments in mice to identify the role of fibroblasts in renal fibrosis. Short-term *Trf1* deletion in renal fibroblasts was not sufficient to trigger kidney fibrosis but was sufficient to induce inflammatory responses, ECM deposition, cell cycle arrest, fibrogenesis, and vascular rarefaction. However, long-term persistent deletion of *Trf1* in fibroblasts resulted in kidney fibrosis accompanied by an elevated urinary albumin-to-creatinine ratio (uACR) and a decrease in mouse survival. These cellular responses lead to the macrophage-to-myofibroblast transition (MMT), endothelial-to-mesenchymal transition (EndMT), and partial epithelial-to-mesenchymal transition (EMT), ultimately causing kidney fibrosis at the humane endpoint (HEP) when the deletion of *Trf1* in fibroblasts is maintained throughout the lifespan of mice. Our findings contribute to a better understanding of the role of dysfunctional telomeres in the onset of the profibrotic alterations that lead to kidney fibrosis.

## Fibroblast telomere dysfunction: key driver of kidney fibrosis

Chronic kidney disease, a condition that can lead to kidney failure and death, affects millions worldwide. It’s characterized by kidney fibrosis, a process where healthy kidney tissue becomes scar tissue. The exact cells that start this process were unknown. Researchers studied the role of a protein called TRF1, which protects the ends of chromosomes, in kidney fibroblasts, cells that make connective tissue. They removed TRF1 from these cells in mice and observed the effects. Results showed that removing TRF1 from fibroblasts increased fibrosis, inflammation, and kidney damage. Specifically, fibroblasts without TRF1 were more likely to change into myofibroblasts, leading to more scar tissue. Study concluded that TRF1 is vital in preventing kidney fibrosis by keeping fibroblasts healthy. This finding improves our understanding of CKD and suggests potential treatments targeting fibroblasts and TRF1 to fight kidney fibrosis.

This summary was initially drafted using artificial intelligence, then revised and fact-checked by the author.

## Introduction

Chronic kidney disease (CKD) has emerged as one of the leading causes of mortality worldwide, affecting more than 10% of the world’s population (between 700 million and one billion people)^1,2^. Renal fibrosis is characterized by a slow deterioration of the functional renal parenchyma, and this sustained kidney dysfunction is associated with irreversible loss of kidney cells and nephrons, a hallmark of CKD, which can ultimately lead to end-stage renal disease (ESRD)^3,4^. Resident fibroblasts are considered as the main cellular sources of scar-forming myofibroblasts responsible for ECM deposition, leading to kidney fibrosis^5,6^.

Telomeres are heterochromatic structures localized at linear chromosome ends that are essential for chromosome stability^7–9^. Telomeres are bound by a specialized complex known as shelterin, which functions in regulating telomere length and protecting telomeres from the DNA damage response (DDR) by masking the chromosome ends from the DNA repair machinery through repression of the ATM and ATR signaling pathways^10^. The shelterin complex is composed of six proteins: telomeric repeat binding factors 1 and 2 (TRF1 and TRF2), TRF1-interacting protein 2 (TIN2), protection of telomeres protein 1 (POT1), POT1-interacting protein (TPP1), and repressor/activator protein 1 (RAP1)^8,9,11^. The telomerase complex is composed of a reverse transcriptase subunit (TERT) and an RNA component (*Terc*), which is used as a template for the de novo addition of telomeric repeats^7^. Telomerase is highly expressed during early embryonic development and in pluripotent stem cells, but its expression is downregulated in the majority of adult cell types, thus leading to telomere shortening associated with cell division and aging. Indeed, telomere shortening has been proposed as one of the hallmarks of aging^11^. Interestingly, a number of diseases are associated with faster telomere shortening with aging due to mutations in telomerase and other telomere components, including pulmonary and kidney fibrosis, known as telomere syndrome^12–14^. Using mouse models, we showed that telomere shortening associated with either telomerase deficiency or telomere dysfunction due to depletion of the TRF1 protein in the shelterin complex was sufficient to induce interstitial fibrosis in the lungs, similar to the findings from human patients carrying mutations in telomere genes. *Trf1* deletion has also been shown to be sufficient to induce pulmonary fibrosis^12,15,16^ and bone marrow failure^17^. Telomere dysfunction is also associated with a loss of renal function^18,19^. In addition, critical telomere shortening in the kidney leads to increased senescence and apoptosis in response to injury^20–22^.

We have previously shown that short telomeres sensitize the kidney to developing fibrosis and that genetic ablation of *Trf1* leads to the acquisition of a mesenchymal phenotype, that has been associated with renal fibrosis, recapitulating human disease^14^. However, the cells of origin of kidney fibrosis associated with telomere dysfunction remain unknown.

In this work, we aimed to address the role of TRF1 in kidney fibroblasts, given its significant role as one of the initiator cells of renal interstitial fibrosis. We showed that short-term *Trf1* deletion in fibroblasts per se does not cause renal fibrosis but rather induces fibrogenesis and inflammation and triggers a cascade of events that result in the transition of fibroblasts to myofibroblasts via the macrophage-to-myofibroblast transition (MMT), partial epithelial-to-mesenchymal transition (EMT), and endothelial-to-mesenchymal transition (EndMT). *Trf1* deletion in fibroblasts contributes to epithelial and endothelial dysfunction and causes hypoxia and G2/M arrest in the kidney. Interestingly, long-term TRF1 depletion causes fibroblasts to acquire an exorbitant mesenchymal phenotype via the MMT, EMT and EndMT, causing extracellular matrix deposition and leading to renal fibrosis, with an increased urine albumin‒creatinine ratio (uACR) and a decline in survival rates.

## Materials and methods

### Mice and animal procedures

**Fig. 1:**
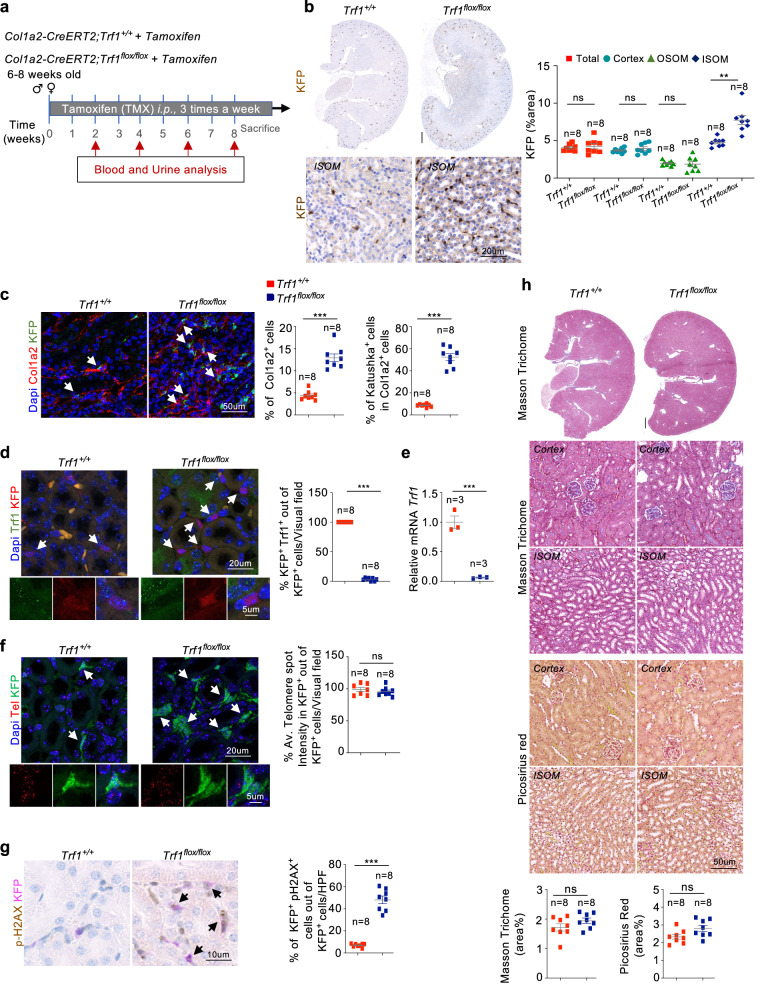
Conditional deletion of *Trf1* in fibroblasts. **a** Schematic of the experimental approach. **b** Representative images of immunostaining and quantification of KFP-positive cells (whole kidney, upper panel; magnified view, lower panel) in *Trf1*^*+/+*^ and *Trf1*^*flox/flox*^ kidneys. Scale bars, 500 μm. **c** Representative images of immunofluorescence staining for Col1a2 (red) and KFP (green) and quantification of the Col1a2^+^ area (whole kidney section) and Col1a2^+^KFP^+^ cells (six to eight visual fields) among Col1a2^+^ cells in *Trf1*^*+/+*^ and *Trf1*^*flox/flox*^ kidneys. **d** Representative images of immunofluorescence staining (six to eight visual fields) for Trf1 (green) and KFP (red) and quantification of the percentage of double KFP^+^TRF1^+^ fibroblasts in *Trf1*^*+/+*^ and *Trf1*^*flox/flox*^ kidneys. **e** Relative mRNA expression of *Trf1* in *Trf1*^*+/+*^ and *Trf1*^*flox/flox*^ kidneys. For all groups, *n* = 3 mice in each group. **f** Representative images (six to eight visual fields) of telomeric immuno-qFISH in KFP^+^ fibroblasts (Cy3Tel probe (red), KFP^+^ cells (green)) and quantification of the mean telomere spot intensity in *Trf1*^*+/+*^ and *Trf1*^*flox/flox*^ kidneys. **g** Representative images of immunostaining (15–20 visual fields) and quantification of p-H2AX^+^ KFP^+^ cells in *Trf1*^*+/+*^ and *Trf1*^*flox/flox*^ kidneys**. h** Representative images (15–20 visual fields) and quantification of Masson’s trichrome and Picrosirius red staining in *Trf1*^*+/+*^ and *Trf1*^*flox/flox*^ kidneys. Scale bars, 500 μm. Nuclei are stained with DAPI (blue). The data are presented as the means ± SEMs. An unpaired, two-tailed Student’s *t* test was used. *p < 0.05, **p < 0.01, and ***p < 0.001. The number of mice analyzed per genotype is *n* = 8, if not indicated.

*Trf1*^*flox/flox*^ mice were generated as previously described^23^. *Trf1*^*flox/flox*^ mice were crossed with transgenic mice expressing *Cre*^*ERT2*^ under the control of the *Col1a2* promoter to conditionally delete *Trf1* in fibroblasts, as well as with transgenic mice harboring the Katushka fluorescent protein (KFP)-encoding gene that contains a stop cassette flanked by lox sequences, the *KFP*^*CAG-lox-STOP-lox*^ allele^24,25^ (Supplementary Fig. 1a). Tamoxifen (TMX) (Sigma–Aldrich, St.Louis, MO, US; T-5648-1G) was intraperitoneally (*i.p*.) injected into six- to eight-week-old male and female *Trf1*^*+/+*^ and *Trf1*^*flox/flox*^ mice three times a week for a duration of 8 weeks to allow the mice to recover between doses (Fig. 1a). Blood and urine samples were collected every two weeks. In another cohort, the mice received tamoxifen three times a week for 8 weeks, followed by once a week until the humane endpoint (HEP) was reached (Fig. 6a). Blood and urine samples were collected every month. The mice were euthanized after 8 weeks and at the HEP, and the kidneys were perfused with ice-cold PBS and harvested. For folic acid (FA)-induced nephropathy, a single intraperitoneal injection of FA at a low dose of 125 mg kg^−1^ body weight or 0.3 M NaHCO_3_ (200 µl) was injected into *Trf1*^*+/+*^ and *Trf1*^*flox/flox*^ mice after an 8-week period of TMA administration (Fig. 7a). Blood was collected on days 0, 2, 7 and 14 after FA injection. The mice were euthanized on day 14, and the kidneys were perfused with cold PBS and harvested. Blood samples were analyzed via the VetScan VS2 Comprehensive Diagnostic Profile Kit, and urine albumin and creatinine levels were analyzed using the Mouse Albumin ELISA Kit (MO-MSAKT) and Creatinine Assay Kit (ab65340), respectively.

All experiments and animal procedures were approved by our Institutional Animal Care and Use Committee (IACUC) (IACUC.022-2018, CBA_03_2019), by the Ethics Committee for Research and Animal Welfare (CEIyBA) (CBA 03-2019-v2) from the Instituto de Salud Carlos III, and by Consejería de Medio Ambiente, Administración Local y Ordenación del Territorio (Comunidad de Madrid) (PROEX 100/19). All experiments and animal procedures were performed in accordance with the guidelines stated in the International Guiding Principles for Biomedical Research Involving Animals, developed by the Council for International Organizations of Medical Sciences (CIOMS). The animals were bred and maintained under specific pathogen-free (SPF) conditions in laminar flow cages with a 12-hour light/dark cycle and were fed *ad libitum* at the CNIO animal facility in accordance with the recommendations of the Federation of European Laboratory Animal Science Associations (FELASA).

### Histopathological analyses, immunohistochemical and immunofluorescence staining

Kidneys were fixed with 4% formaldehyde, embedded in paraffin and OCT (Tissue-Tek, 4583), and cut into 5–7 μm sections for the histopathological evaluation, immunohistochemistry (IHC) or immunofluorescence (IF) staining. Hematoxylin and eosin (H&E) staining was performed for the histopathological evaluation. Masson’s trichome and Picosirius red stainings were used to evaluate collagen deposition, and periodic acid–Schiff plus diastase (PAS + D) was used to evaluate tubular damage. Formalin-fixed, paraffin-embedded kidney sections were deparaffinized and rehydrated. Antigen retrieval was performed at 98 °C for 15 min in 10 mM citrate buffer (pH 6), after which the samples were incubated with primary antibodies and appropriate horseradish peroxidase (HRP)-conjugated secondary antibodies or Alexa Fluor-conjugated antibodies.

The following antibodies were used for immunohistochemistry: rabbit turbo-RFP (EVN-AB233, Evrogen; 1:3000), mouse smooth muscle actin (Clone 1A4, IR611, Dako; ready to use), rabbit F4/80 (BLR209K, A700-209, Bethyl; 1:100), rabbit phospho-Histone H3 (ser10, 06-570, Millipore; 1:500), mouse phospho-Histone H2AX (Ser 139, 05-636, Millipore; 1:25000), rabbit NF-kB p65 (Clone C-20, Santa Cruz Biotechnology, 1:300), mouse IL-6 (Clone 1.2-2B11-2G10, ab9324, Abcam, 1:3000), rabbit Jak2 (phospho Y1007 + Y1008 (Clone 1E132, ab32101, Abcam, 1:200), rabbit phospho-Stat3 (Tyr705) (Clone D3A7, 9145, Cell Signaling; 1:50), rabbit CD31 (Clone EPR17259, ab182981, Abcam; 1:2000), rat Hif-1 alpha (Clone 343B, CNIO Monoclonal Antibodies Core Unit, Madrid, Spain, 1:30), rabbit Collagen type 1 (AB765P, Sigma–Aldrich; 1:600), rabbit Vegfr2 (Clone 55B11, 2479, Cell Signaling; 1:200), rabbit Vimentin (Clone D21H3, 5741, Cell Signaling; 1:50), mouse Glut1 (Clone SPM498, ab40084, Abcam, 1:250).

For immunofluorescence staining, rabbit turbo-RFP (EVN-AB233, EVROGEN, 1:200), mouse COL1A2 (Clone E-6, SC-393573, Santa Cruz Biotechnology, 1:400), rat TRF1 (Clone 572 C, CNIO Monoclonal Antibodies Core Unit, Madrid, Spain, 1:400), goat 53BP1 (PLA0303-100UL, Bethyl; 1:400), rat F4/80 (MCA497; AbD Serotec 1:400), mouse α-SMA-Cy3 (C6198, Sigma–Aldrich; 1:400), rat E-cadherin (Clone DECMA-1, ab11512, Abcam; 1:200), rabbit Snail+Slug (ab180714, Abcam; 1:200), rat Cd31 (Clone MEC 13.3, 553370, BD Biosciences; 1:400), and mouse Cd206 (Clone MR5D, MCA2235F, Bio-Rad; 1:200) antibodies were used. For the dextran permeability assay, the mice were injected i.v. with 50 µl of 2 mg/ml 70 kDa FITC-dextran (Sigma–Aldrich, 90718) before sacrifice, and the kidney sections were directly visualized as formalin-fixed, paraffin-embedded sections. The sections were blocked with 3% bovine serum albumin (BSA) in PBS for 2 h and incubated overnight with anti-CD31 (Clone MEC 13.3, 553370, BD Biosciences; 1:400) and rabbit turbo-RFP (EVN-AB233, EVROGEN; 1:400) antibodies and subsequently with anti-rat Alexa Fluor 555 (A21434, Invitrogen; 1:400) and anti-rabbit Alexa Fluor 647 (A21443, Invitrogen; 1:400) antibodies for 2 h. DAPI was used for nuclear staining. All the immunohistochemistry images were scanned with a Mirax 3D Histech scanner, whereas the immunofluorescence images were captured with a TCS SP8 STED 3X (Leica Microsystems) microscope.

The percentages of positively stained areas detected using immunohistochemistry and immunofluorescence staining were quantified with NIH ImageJ (v1.52n) or Qupath (v0.4.1) software.

### Telomere q-FISH analyses

The telomere quantitative-FISH determination of paraffin-embedded tissue sections was performed as described previously^12,14^. After deparaffinization and rehydration, the tissues were subjected to antigen retrieval in 10 mM sodium citrate buffer, and permeabilization was performed in 0.5% Triton X-100 in PBS for 3 h. Next, the tissues were washed 3 × 5 min with 1X PBS, fixed with 4% formaldehyde for 5 min, washed 3 × 5 min with PBS and dehydrated in a 70%–90%–100% ethanol series (5 min each). After 10 min of air drying, 30 μl of the telomere probe mixture (10 mM Tris-Cl (pH 7), 25 mM MgCl_2_, 9 mM citric acid, 82 mM Na_2_HPO_4_, 70% deionized formamide (Sigma), 0.25% blocking reagent (Roche) and 0.5 μg ml^−1^ telomeric PNA probe (Panagene)) was added to each slide. A coverslip was added, and the sections were incubated for 3 min at 85 °C and for an additional 2 h at room temperature in a wet chamber in the dark. The sections were washed twice for 15 min each with 10 mM Tris-Cl (pH 7) and 0.1% BSA in 70% formamide with vigorous shaking and then three times for 5 min each with TBS containing 0.08% Tween 20. The sections were blocked with 3% BSA for 2 h and incubated with a rabbit turbo-RFP (EVN-AB233, EVROGEN, 1:200) antibody overnight at 4 °C for immuno-q-FISH. The sections were washed with PBS (0.5% tween) three times for 15 min each, incubated with an Alexa Fluor 647-conjugated anti-rabbit secondary antibody (A21443, Invitrogen; 1:400) for 2 hours and mounted with Vectashield medium (Vector). DAPI was used for nuclear staining. Using the same protocol, immuno-telomere-q-FISH with the DNA damage marker 53BP1 (PLA0303-100UL, Bethyl; 1:400) was performed to identify telomeric induced foci (TIF) in rabbit turbo-RFP (EVN-AB233, EVROGEN, 1:200)-positive cells. Confocal images were acquired as stacks every 1 μm for a total of 5 μm using a Leica SP8 confocal microscope. The telomere signal intensity was quantified using Definiens software.

### Cell sorting

*Trf1*^*+/+*^ and *Trf1*^*flox/flox*^ kidneys were collected in ice-cold PBS, minced, and digested with 2.5 mg/ml type I collagenase (Thermo Fisher, 17100017), 2.5 mg/ml dispase II (Thermo Fisher, 17105041), and 50 ng/ml DNase I (Roche, 10104159001) at 37 °C for 20 min to create a homogeneous cell suspension to isolate the fibroblast subpopulation. The cell suspensions were passed through a 70 μm filter (Cultek, 45352350) to remove any undigested tissue. The cell suspensions and blood samples were incubated for 10 min on ice in blood erythrocyte lysis buffer (Qiagen, 79217) to remove erythroid cells. Before analysis, the cell suspensions were treated with 0.04% ultrapure BSA in PBS (Thermo Fisher, AM216). The cells were incubated at 4 °C for 30 min with anti-mouse CD29-PE (clone: HMB1-1, 1:00) (Milteneyi Biotec, 130-102-994) and anti-t-RFP (EVN-AB233, Evrogen; 1:00) for fibroblast isolation. An Alexa Fluor 647-conjugated chicken anti-rabbit antibody (A21443; Invitrogen; 1:400) was used as the secondary antibody for t-RFP. Isolated cells were mixed with NEBNext^®^ Cell Lysis Buffer (New England Biolabs, E6428A) and murine RNase Inhibitor (New England Biolabs, E6429A) in DNA LoBIND tubes (Eppendorf, 0030108051), and then analyzed using a BD FACSAria IIu cell sorter (BD Biosciences) at the Flow Cytometry Core Unit, CNIO.

### RNA-seq and analysis

A total of 500 FACS-sorted cells from both *Trf1*^*+/+*^ and *Trf1*^*flox/flox*^ kidneys were processed with the NEBNext Single Cell/Low Input RNA Library Prep Kit (New England Biolabs, E6420) according to the manufacturer’s instructions. Briefly, extracts obtained from samples suspended in cell lysis buffer were subjected to oligo(dT)-primed reverse transcription with a template-switching reaction. Double-stranded cDNA production was performed via limited-cycle PCR. The sequencing libraries were completed with the NEBNext Ultra II FS DNA Library Prep Kit for Illumina (New England Biolabs, E7805) and subsequently analyzed on an Illumina instrument (see below) according to the manufacturer’s protocols. (These libraries are non-directional or non-stranded.) The resulting purified cDNA libraries were applied to an Illumina flow cell for cluster generation and sequenced on an Illumina NextSeq 550 instrument (with v2.5 reagent kits) according to the manufacturer’s protocols. Raw images generated by the sequencer were submitted for analysis, per-cycle base calling and quality score assignment with Illumina’s real-time analysis (RTA)-integrated primary analysis software. BCL (base call) binary files were subsequently converted to FASTQ format with the Local Run Manager GenerateFASTQ Analysis Module (Illumina). Read adaptors and poly(A) tails were removed with the command ‘bbduk.sh’ according to the Lexogen recommendations. Processed reads were then analyzed with the Nextpresso pipeline^26^. Sequencing quality was checked with FastQC v0.11.7 (https://www.bioinformatics.babraham.ac.uk/projects/fastqc/). Reads were aligned to the mouse reference genome (GRCm38) with TopHat (v2.0.10)^27^ using Bowtie (v1.0.0)^28^ and SAMtools (v0.1.19)^29^ (--librarytype: fr-second strand in TopHat), allowing three mismatches and twenty multihits. Read counts were obtained with HTSeq-count (v0.6.1)^30^ using the mouse gene annotation from GENCODE (GRCm38; vM20 Ensembl 95). GSEAPreranked was used to perform GSEA for several gene signatures with a preranked gene list, setting 1000 gene set permutations^31^. Only those gene sets with significant enrichment levels (FDR *q* value < 0.25) were considered. The RNA-seq files with raw and processed data are available from Gene Expression Omnibus (GEO) with the following ID: GSE232874.

### RT‒qPCR and ELISA

Nearly 40,000 FACS-isolated KFP^+^ cells from the *Trf1*^*+/+*^ and *Trf1*^*flox/flox*^ kidneys were collected in RLT buffer and processed for total RNA extraction with the RNeasy Micro Kit (Qiagen) according to the manufacturer’s instructions. cDNA was synthesized with 1 μg of total RNA, cDNA synthesis mix (BioMake), and oligo-dT primers. Gene expression was measured via a real-time PCR assay (BioMake) and a 7900HT real-time PCR system (Applied Biosystems). The relative amount of mRNA to the internal control was calculated as 2^∆CT^, in which ∆CT = ∆CT_experimental_ − ∆CT_control_. The genes and primers used are listed in Supplementary Table 1 (F: forward; R: reverse primer). Approximately 20,000 FACS-isolated KFP^+^ cells from *Trf1*^*+/+*^ and *Trf1*^*flox/flox*^ kidneys were collected under sterile conditions in PBS. The cells were plated in dishes coated with 1% gelatin and cultured in DMEM (Thermo Fischer Scientific, 11966025) supplemented with 20% FBS (Sigma–Aldrich, F7524) and 100 IU ml^–1^ penicillin/streptomycin (Thermo Fischer Scientific, 15070063) for 48 hours. The supernatant was collected and used for ELISAs. Mouse TNF-alpha, IL1-beta, IL-6 and pSTAT3 levels were determined using mouse TNF-α quantitative ELISA kit (R and D Systems, MTA00B), an IL-1 beta/IL-1F2 quantitative ELISA kit (R and D Systems, MLB00C), a mouse IL-6 quantitative ELISA kit (R and D Systems, M6000B) and a PathScan^®^ phospho-Stat3 (Tyr705) sandwich ELISA kit (Cell Signaling, 7300), respectively, according to the manufacturers’ instructions.

### Statistics

No statistical methods were used to predetermine sample sizes, but our sample sizes are similar to those reported previously^32^. The mice were randomly allocated to groups, and the investigators were blinded to the group allocations. Our sample sizes corresponded to the number of mice used for each experiment, as indicated. For single IHC staining, whole kidneys were quantified. For double staining, the whole scanned kidney section comprising 15–20 areas were quantified. For the immunofluorescence analysis, 6 to 10 images were collected from each individual. The data distribution was assumed to be normal, but this parameter was not formally tested. No data points or mice were excluded from the analyses. When comparisons were performed between two experimental groups, unpaired two-tailed Student’s *t* test was used. Overall survival was assessed via Kaplan–Meier survival curves using the log rank (Mantel–Cox) test. Statistical significance was defined as *P* < 0.05.

## Results

### Transient *Trf1* deletion in renal fibroblasts does not affect kidney function or cause kidney fibrosis but promotes fibrogenesis and inflammation

We induced short-term telomere dysfunction in fibroblasts by genetically deleting the shelterin component *Trf1* in these cells to explore whether telomere dysfunction in kidney fibroblasts contributes to kidney fibrosis. After an 8-week regimen of TMX treatment, KFP^+^ cells (fibroblast-expressing cells) were detected in the kidney interstitium, and a significant increase in the inner stripe of the outer medulla (ISOM) was detected in *Trf1*^*lox/lox*^ mice (Fig. 1b). However, no alterations in the percentage of KFP^+^ cells in the cortex or the outer stripe of the medulla (OSOM) were detected between *Trf1*^*lox/lox*^ and *Trf1*^*+/+*^ mice (Fig. 1b). Consequently, we focused on the effect of TRF1 depletion in the ISOM region of the kidneys, where the greatest number of KFP+ cells was detected in *Trf1*^*lox/lox*^ and *Trf1*^*+/+*^ mice. A 3-fold increase in global Col1a2^+^ cells, with all KFP^+^ cells positive for Col1a2^+^, and a 6-fold increase in Col1a2^+^KFP^+^ cells were observed in *Trf1*^*lox/lox*^ mice compared to *Trf1*^*+/+*^ mice (Fig. 1c). Immunofluorescence staining with anti-TRF1 and anti-t-RFP antibodies revealed that all KFP^+^ cells in the kidneys of *Trf1*^*lox/lox*^ mice were negative for TRF1 (Fig. 1d). Furthermore, real-time qPCR (RT‒qPCR) analysis confirmed the fibroblast-specific deletion of *Trf1* in *Trf1*^*flox/flox*^ mice (Fig. 1e).

We conducted telomere q-FISH to quantify the mean telomere intensity in KFP^+^ cells and assess the impact of *Trf1* deletion on the telomere length^12^. No significant difference in telomere fluorescence was detected between *Trf1*^*flox/flox*^ and *Trf1*^*+/+*^ mice (Fig. 1f), although a 7-fold increase in the percentage of KFP^+^p-H2AX ^+^ cells was detected in *Trf1*^*flox/flox*^ mice (Fig. 1g). Notably, no alterations in body weight, kidney-to-body weight ratios, or markers of renal dysfunction, such as blood creatinine and blood urea nitrogen (BUN) levels and the urinary albumin-to-creatinine ratio (uACR), were detected at 8 weeks after *Trf1* deletion in fibroblasts (Supplementary Fig. 1b–f). Consistent with these findings, Masson’s trichrome and Picosirius red staining revealed no signs of cortical or interstitial fibrosis in the kidneys of *Trf1*^*flox/flox*^ mice compared with those of *Trf1*^*+/+*^ mice (Fig. 1h). All the other organs studied, including the liver, lung and heart, did not display histological abnormalities (Supplementary Fig. 1g).

**Fig. 2:**
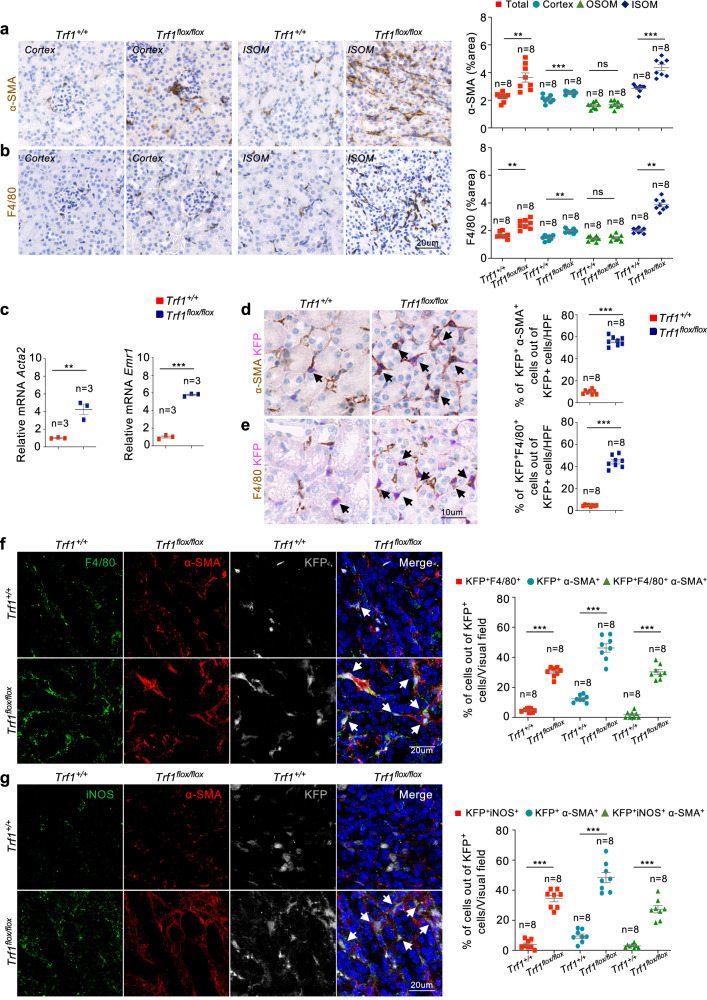
Deletion of Trf1 in fibroblasts induces fibrogenesis and inflammation coincident with the macrophage-to-mesenchymal transition (MMT). Representative images of immunostaining (15–20 visual fields) and quantification of **a** α-SMA and **b** F4/80 expression in *Trf1*^*+/+*^ and *Trf1*^*flox/flox*^ kidneys. **c** Relative mRNA expression of *Acta2* and *Emr1* in *Trf1*^*+/+*^ and *Trf1*^*flox/flox*^ kidneys. For all groups, *n* = 3 mice in each group. **d** Representative images of immunostaining (15–20 visual fields) and quantification of (**d**) α-SMA^+^KFP^+^ and (**e**) F4/80^+^KFP^+^ cells in *Trf1*^*+/+*^ and *Trf1*^*flox/flox*^ kidneys. **f** Representative images (six to eight visual fields) and quantification of immunolabeling for F4/80, α-SMA and KFP in *Trf1*^*+/+*^ and *Trf1*^*flox/flox*^ kidneys. **g** Representative images (six to eight visual fields) and quantification of immunolabeling for iNOS, α-SMA and KFP in *Trf1*^*+/+*^ and *Trf1*^*flox/flox*^ kidneys. Nuclei are stained with DAPI (blue). The data are presented as the means ± SEMs. An unpaired, two-tailed Student’s *t* test was used. *p < 0.05, **p < 0.01, and ***p < 0.001. The number of mice analyzed per genotype is n = 8, if not indicated.

As an indicator of myofibroblast activation and fibrosis, we observed increased numbers of *α*-SMA^+^ myofibroblasts in *Trf1*^*flox/flox*^ mice, indicating the transition of fibroblasts to myofibroblasts as a consequence of *Trf1* deletion (Fig. 2a). Increased numbers of F4/80^+^ macrophages were also observed in *Trf1*^*flox/flox*^ mice, indicating increased inflammation (Fig. 2b). RT‒qPCR analysis further confirmed the upregulation of *Acta2* (which encodes αSMA) and *Emr1* (which encodes F4/80) mRNA levels in *Trf1*^*flox/flox*^ mice compared with those in *Trf1*^*+/+*^ mice (Fig. 2c).

### *Trf1* deletion in renal fibroblasts induces the macrophage-to-myofibroblast transition

Next, we examined the expression of α-SMA and F4/80 in combination with Katushka (KFP, Cre-reporter) to specifically detect TRF1-deleted cells in the interstitium of the kidneys. Immunostaining revealed 6- and 9-fold increases in the numbers α-SMA^+^KFP^+^ and F4/80^+^KFP^+^ cells, respectively, in *Trf1*^*flox/flox*^ mice, indicating increased ECM levels and inflammation in *Trf1*-deleted cells (Fig. 2d, e). The MMT has been identified as a mechanism underlying chronic inflammation leading to progressive fibrosis. Upon injury, monocytes/macrophages infiltrate the renal tissue and undergo a transition to myofibroblasts^33–35^. We assessed this process by performing triple immunostaining to identify KFP^+^ cells undergoing the MMT based on the coexpression of F4/80 and *α*-SMA in cells. These KFP^+^F4/80^+^*α*-SMA^+^ cells accounted for approximately 27% of the total KFP^+^ fibroblast population in the *Trf1*^*flox/flox*^ mice compared with 1.8% in the *Trf1*^*+/+*^ mice (Fig. 2f). Furthermore, we explored the subtypes of macrophages contributing to the MMT by costaining for α-SMA with the macrophage subfamily markers iNOS (M1 macrophages) and CD206 (M2 macrophages)^34,36^. A significant 9-fold increase in the percentage of KFP^+^iNOS^+^*α*-SMA^+^ cells was observed, whereas the percentage of KFP^+^CD206^+^*α*-SMA^+^ cells remained unchanged in *Trf1*^*flox/flox*^ mice compared with *Trf1*^*+/+*^ mice, suggesting that predominantly M1-type macrophages undergo the MMT upon *Trf1* deletion (Fig. 2g; Supplementary Fig. 1h).

### Renal fibroblasts lacking *Trf1* upregulate Tgf-β signaling and the partial EMT pathway

**Fig. 3:**
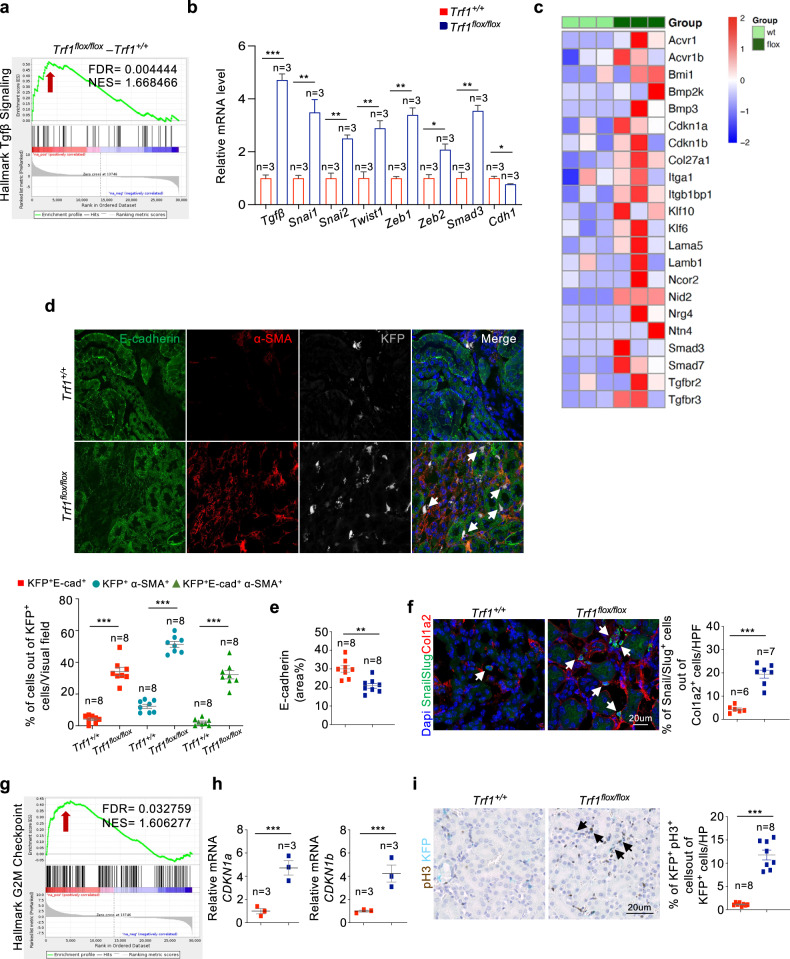
Fibroblast-specific TRF1 deletion upregulates Tgfβ signaling and causes cell cycle arrest at G2/M phase. **a** GSEA enrichment plots of the hallmark gene dataset showing the enrichment of Tgfβ signaling in the kidneys of *Trf1*^*+/+*^ and *Trf1*^*flox/flox*^ mice. For all groups, *n* = 3 mice in each group. **b** Relative mRNA expression of *Tgfβ*, *Snai1*, *Snai2*, *Twist1*, *Zeb1*, *Zeb2*, *Smad3* and *Cdh1* in the kidneys of *Trf1*^*+/+*^ and *Trf1*^*flox/flox*^ mice. For all groups, *n* = 3 mice in each group. **c** Heatmap of the RNA-seq results showing differences in the expression of Tgfβ signaling- and fibrosis-related genes in the kidneys of *Trf1*^*+/+*^ and *Trf1*^*flox/flox*^ mice. **d** Representative images (six to eight visual fields) and quantification of immunolabeling for E-cadherin, α-SMA and KFP in *Trf1*^*+/+*^ and *Trf1*^*flox/flox*^ kidneys. **e** Quantification of E-cadherin expression in the kidneys of *Trf1*^*+/+*^ and *Trf1*^*flox/flox*^ mice. For all groups, *n* = 3 mice in each group. **f** Representative images (six to eight visual fields) and quantification of immunolabeling for Snail/Slug and Col1a2 in *Trf1*^*+/+*^ and *Trf1*^*flox/flox*^ kidneys. Nuclei are stained with DAPI (blue). *Trf1*^*+/+*^ mice *n* = 6; *Trf1*^*flox/flox*^ mice *n* = 7. **g** GSEA enrichment plots of the hallmark gene dataset showing the enrichment of the G2/M checkpoint in the kidneys of *Trf1*^*+/+*^ and *Trf1*^*flox/flox*^ mice. For all groups, *n* = 3 mice in each group. **h** Relative mRNA expression of *Cdkn1a and Cdkn1b* in the kidneys of *Trf1*^*+/+*^ and *Trf1*^*flox/flox*^ mice. For all groups, *n* = 3 mice in each group. **i** Representative images of immunostaining (15–20 visual fields) and quantification of pH3^+^KFP^+^ cells in *Trf1*^*+/+*^ and *Trf1*^*flox/flox*^ kidneys. Nuclei are stained with DAPI (blue). The data are presented as the means ± SEMs. An unpaired, two-tailed Student’s *t* test was used. *p < 0.05, **p < 0.01, and ***p < 0.001. The number of mice analyzed per genotype is *n* = 8, if not indicated.

Transforming growth factor-β (*Tgf-β*) is generally considered the principal mediator of the initiation and progression of renal fibrosis in various diseases via the EMT^3,37–39^. We used the distinct cell surface markers CD29 and KFP to isolate fibroblast subsets from the kidneys of *Trf1*^*flox/flox*^ and *Trf1*^*+/+*^mice using flow cytometry and to delineate the consequences of *Trf1* deletion in fibroblasts (Supplementary Fig. 2a, b). We subsequently performed RNA sequencing to comprehensively elucidate the transcriptomic alterations, as well as RT‒qPCR and ELISA to validate these changes. Overall, gene set enrichment analysis (GSEA) revealed a significantly greater number of enriched gene sets in *Trf1*^*flox/flox*^ mice than in *Trf1*^*+/+*^ mice **(**Supplementary Fig. 2c). GSEA confirmed the enrichment of the Tgf-β signature (NES = 1.6) in *Trf1*^*flox/flox*^ mice compared with *Trf1* mice (Fig. 3a). RT‒qPCR analysis confirmed elevated expression of *Tgf-β*, *Snai1, Snai2*, *Twist1*, *Zeb1*, *Zeb2*, and *Smad3* and the downregulation of *Cdh1* in *Trf1*^*flox/flox*^ mice (Fig. 3b). Furthermore, RNA-seq data confirmed that various *Tgf-β* signaling- and fibrosis-related genes were upregulated in *Trf1*^*flox/flox*^ mice compared with *Trf1*^*+/+*^ mice (Fig. 3c). As a method to elucidate the role of *Trf1* deletion and the characteristics of KFP^+^ interstitial cells in the EMT, kidney samples from *Trf1*^*flox/flox*^ and *Trf1*^*+/+*^ mice were triple immunolabeled to identify KFP^+^ cells undergoing the EMT based on the co-expression of E-cadherin (E-cad) and *α*-SMA. A significant decrease in both E-cadherin staining and mRNA levels was detected, accompanied by an 8-fold increase in the number of KFP^+^E-cad^+^ cells in *Trf1*^*flox/flox*^ mice (Fig. 3d, e). The population of cells undergoing the EMT (KFP^+^E-cad^+^*α*-SMA^+^) accounted for approximately 32% of the total KFP^+^ fibroblast population in the *Trf1*^*flox/flox*^ mice (Fig. 3d). The colocalization of Snai/Slug and Col1a2 in interstitial myofibroblasts was 4-fold greater in *Trf1*^*flox/flox*^ mice than in *Trf1*^*+/+*^ mice, suggesting that the EMT program is activated in *Trf1*^*flox/flox*^ mice upon *Trf1* deletion in renal fibroblasts **(**Fig. 3f).

### *Trf1* deletion in renal fibroblasts results in G2/M arrest

Severe acute kidney injury (AKI) has been suggested to trigger tubular cell cycle arrest in G2/M phase, accompanied by the activation of the SASP^32^. Consistent with these findings, our GSEA confirmed the enrichment of G2M checkpoint pathways (NES = 1.6) alongside the upregulation of *Cdkn1a* and *Cdkn1b* mRNA levels and a 10-fold increase in the number of KFP^+^pH3^+^ cells in *Trf1*^*flox/flox*^ mice compared with those in *Trf1*^*+/+*^ mice (Fig. 3g–i). Moreover, approximately 60% of the KFP^+^pH3^+^ cells displayed scattered foci of pH3^+^, indicating a prolonged G2/M phase of the cell cycle.

### *Trf1* deletion in renal fibroblasts induces an inflammatory response

**Fig. 4:**
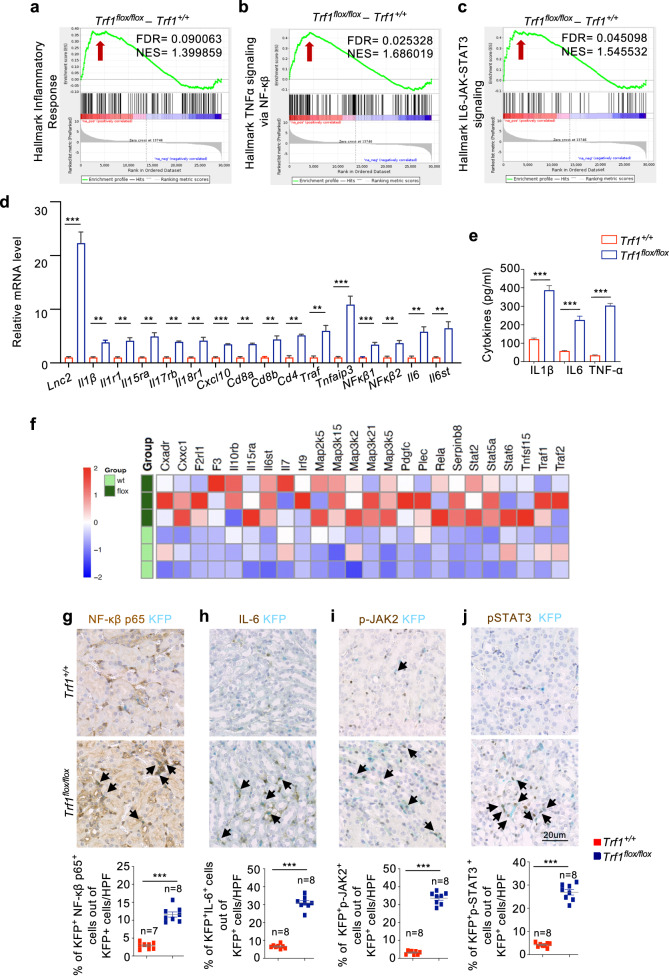
Fibroblast-specific deletion of *Trf1* increases inflammation. GSEA enrichment plots of the hallmark gene dataset revealed the enrichment of (**a**) the inflammatory response, **b** TNFα signaling via NF-κβ, and **c** IL6-JAK-STAT3 signaling in the kidneys of *Trf1*^*+/+*^ and *Trf1*^*flox/flox*^ mice. For all groups, *n* = 3 mice in each group. **d** Relative mRNA expression of *Lnc2*, *IL1β, IL1r1, IL15rα, IL17rb, IL18r1, Cxcl10, Cd8a*, *Cd8b, Cd4, Traf1*, *Tnfaip3*, *NF-κβ1, NF-κβ2*, *IL6* and *IL6st in* the kidneys of *Trf1*^*+/+*^ and *Trf1*^*flox/flox*^ mice. For all groups, *n* = 3 mice in each group. **e** ELISAs of IL-1β, IL6 and TNFα levels in the kidneys of *Trf1*^*+/+*^ and *Trf1*^*flox/flox*^ mice. For all groups, *n* = 3 mice in each group. **f** Heatmap of the RNA-seq results showing differences in the expression of inflammatory genes and genes in associated targeted pathways in the kidneys of *Trf1*^*+/+*^ and *Trf1*^*flox/flox*^ mice. Representative images of immunostaining (15–20 visual fields) and quantification of (**g**) NF-κβ p65^+^KFP^+^, (**h**) IL6^+^KFP^+^, (**i**) p-JAK2^+^KFP^+^, and (**j**) p-STAT3^+^KFP^+^ cells in the kidneys of *Trf1*^*+/+*^ and *Trf1*^*flox/flox*^ mice. The data are presented as the means ± SEMs. An unpaired, two-tailed Student’s *t* test was used. *p < 0.05, **p < 0.01, and ***p < 0.001. The number of mice analyzed per genotype is *n* = 8, if not indicated.

Inflammation is considered a pivotal factor in kidney fibrosis and is characterized by intricate crosstalk between resident fibroblasts and infiltrating cells^40^. Our GSEA revealed a significant enhancement of the inflammatory response (NES: 1.39), along with prominent activation of major inflammatory pathways, TNFα via NF-κβ (NES: 1.68), IL-6-JAK-STAT3 (NES: 1.54), and IL2-STAT5 (NES: 1.45) signaling, the IFN-γ response (NES: 1.38), and the complement cascade (NES: 1.21) in *Trf1*^*flox/flox*^ mice compared with *Trf1*^*+/+*^ mice (Fig. 4a–c**;** Supplementary Fig. 2d–f). The RT‒qPCR analysis of the transcript levels of proinflammatory genes, such as *Lcn2, IL1β, IL1R1, IL17rb*, *lL18r1*, *Cxcl10, CD8a*, *CD8b*, *CD4, Traf1*, *Tnfaip3, NF-κβ1, NF-κβ2, IL-6 and IL6st*, revealed their significant upregulation in *Trf1*^*flox/flox*^ mice compared with *Trf1* mice (Fig. 4d). The deletion of *Trf1* substantially increased the secretion of IL-1β, TNFα, IL-6 and pSTAT3 in *Trf1*^*flox/flox*^ mice compared with *Trf1*^*+/+*^ mice (Fig. 4e**;** Supplementary Fig. 2g). RNA-seq revealed the upregulation of target genes associated with various inflammatory pathways in *Trf1*^*flox/flox*^ mice compared with *Trf1*^*+/+*^ mice (Fig. 4f). Next, we validated these results by performing multiplex immunolabeling of the NF-κβ p65 subunit, IL6, p-Jak2, and p-Stat3 in combination with t-RFP. We detected 3.8-, 4.6-, 10- and 6-fold increases in the numbers of NF-κβp65^+^KFP^+^, IL6^+^KFP^+^, p-Jak2^+^KFP^+^, and p-Stat3^+^KFP^+^ cells, respectively, in *Trf1*^*flox/flox*^ mice compared with those in *Trf1*^*+/+*^ mice (Fig. 4g–j).

### *Trf1* deletion in renal fibroblasts induces capillary rarefaction, the EndMT and hypoxia

**Fig. 5:**
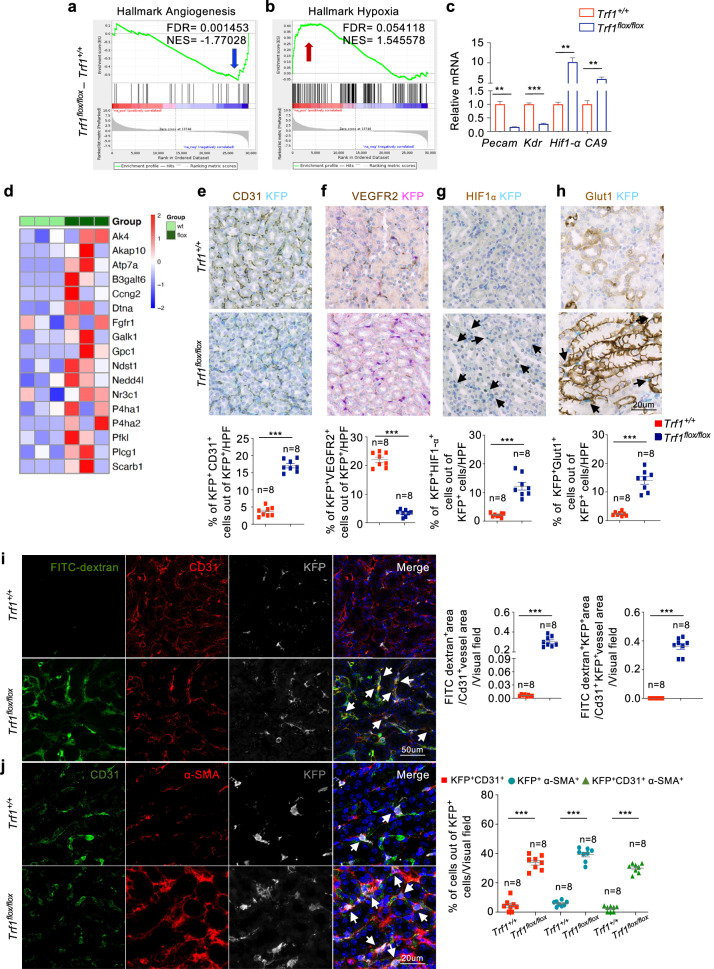
Fibroblast-specific deletion of *Trf1* induces capillary rarefaction, the EndoMT and hypoxia. GSEA enrichment plots of the hallmark gene dataset showed decreases in (**a**) angiogenesis and (**b**) hypoxia in the kidneys of *Trf1*^*+/+*^ and *Trf1*^*flox/flox*^ mice. For all groups, *n* = 3 mice in each group. **c** Relative mRNA expression of *Pecam1, Kdr, HIF-1α and CA9* in the kidneys of *Trf1*^*+/+*^ and *Trf1*^*flox/flox*^ mice. For all groups, *n* = 3 mice in each group. **d** Heatmap of the RNA-seq results showing differences in the expression of angiogenesis- and hypoxia-related genes in the kidneys of the *Trf1*^*+/+*^ and *Trf1*^*flox/flox*^ mice. Representative images of immunostaining (15–20 visual fields) and quantification of (**e**) CD31^+^KFP^+^, (**f**) Vegfr2^+^KFP^+^, (**g**) HIF-1α^+^ and KFP^+^, and (**h**) Glut1^+^ and KFP^+^ cells in the *Trf1*^*+/+*^ and *Trf1*^*flox/flox*^ kidneys. **i** Representative images (six to eight visual fields) and quantification of immunolabeling for FITC-dextran, CD31 and KFP in *Trf1*^*+/+*^ and *Trf1*^*flox/flox*^ kidneys. **j** Representative images (six to eight visual fields) and quantification of immunolabeling for CD31, α-SMA and KFP in *Trf1*^*+/+*^ and *Trf1*^*flox/flox*^ kidneys. Nuclei are stained with DAPI (blue). The data are presented as the means ± SEMs. An unpaired, two-tailed Student’s *t* test was used. *p < 0.05, **p < 0.01, and ***p < 0.001. The number of mice analyzed per genotype is n = 8, if not indicated.

The EndMT is a cellular transdifferentiation program in which endothelial cells partially lose their endothelial identity and acquire mesenchymal-like features^32^. We scrutinized our GSEA data to explore whether *Trf1* deletion in fibroblasts contributes to injury-induced endothelial dysfunction in kidney fibrosis and observed a significant reduction in angiogenesis (NES: 1.77), marked by the downregulation of *Pecam1* and *Kdr* mRNA levels, and an increase in hypoxia (NES: 1.54), accompanied by elevated mRNA levels of *Hif1-α* and *CA9*, in *Trf1*^*flox/flox*^ mice compared with *Trf1* mice (Fig. 5a–c). The RNA-seq data revealed the upregulation of target genes associated with angiogenesis and the hypoxia pathway in the *Trf1*^*flox/flox*^ compared with the *Trf1*^*+/+*^ mice (Fig. 5d). *Trf1*^*flox/flox*^ mice presented 3- and 5-fold decreases in the numbers of CD31^+^ and Vegfr2^+^ cells and 5- and 7-fold increases in the numbers of CD31^+^KFP^+^ and Vegfr2^+^KFP^+^ cells (Fig. 5e, f; Supplementary Fig. 3a, b). Conversely, 5- and 9-fold increases in the numbers of HIF1α^+^KFP^+^ and Glut1^+^ KFP^+^ cells were evident in *Trf1*^*flox/flox*^ mice compared with *Trf1*^*+/+*^ mice (Fig. 5g, h). *Trf1*^*flox/flox*^ and *Trf1*^*+/+*^ mice were systemically administered high-molecular-weight FITC-conjugated dextran (70 kDa) to investigate whether *Trf1* deletion in fibroblasts impaired endothelial cell‒cell junctions, possibly affecting vascular permeability. Interestingly, we observed significantly greater amounts of interstitial dextran in the kidneys of *Trf1*^*flox/flox*^ mice than in those of *Trf1*^*+/+*^ mice, suggesting increased vascular leakage upon *Trf1* deletion (Fig. 5i). Furthermore, we investigated whether *Trf1* deletion-induced endothelial dysfunction leads to the activation of an EndMT program. We performed confocal imaging of kidney tissues triple-immunolabeled for CD31, *α*-SMA and KFP to identify the KFP^+^ cells that were undergoing the EndMT. Notably, these KFP^+^CD31^+^*α*-SMA^+^ cells accounted for approximately 30% of the total KFP^+^ fibroblast population in *Trf1*^*flox/flox*^ mice compared with 2.32% in *Trf1*^*+/+*^ mice, suggesting that *Trf1* deletion in fibroblasts affects endothelial cells, leading to the EndMT (Fig. 5j).

### Persistent long-term *Trf1* deletion in renal fibroblasts leads to renal fibrosis

**Fig. 6:**
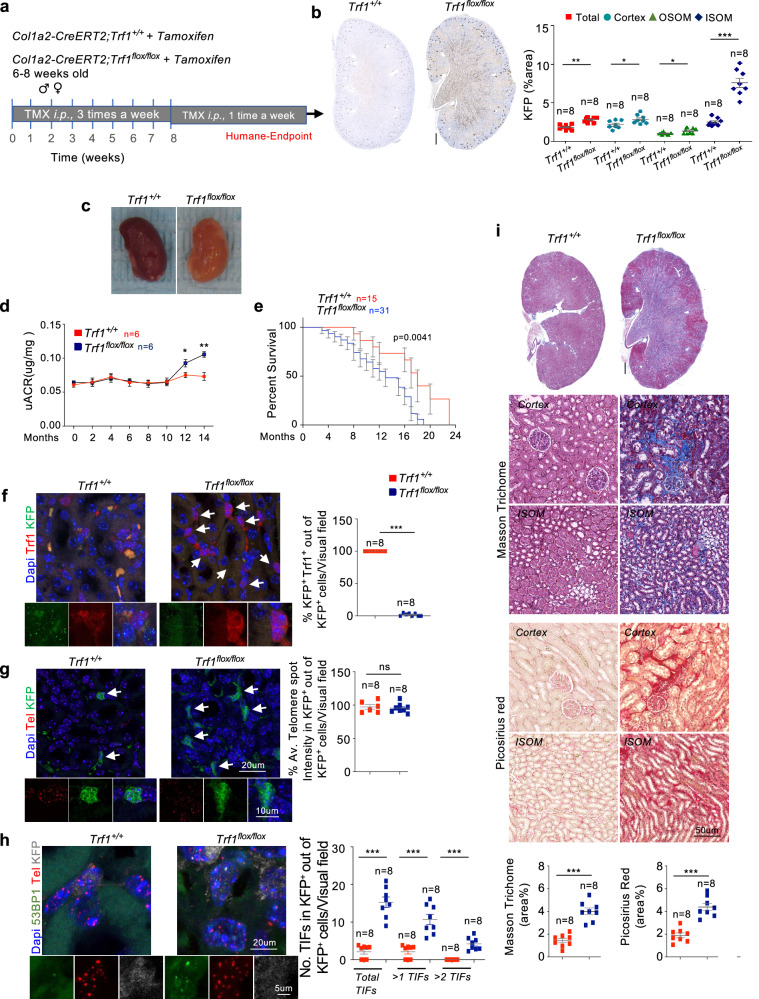
Persistent long-term fibroblast-specific deletion of *Trf1* induces kidney fibrosis at the HEP. **a** Schematic of the experimental approach. **b** Representative images of immunostaining and quantification of KFP-positive cells (whole kidney, upper panel; magnified view, lower panel) in *Trf1*^*+/+*^ and *Trf1*^*flox/flox*^ kidneys. Scale bars, 500 μm. **c** Macroscopic appearance of the kidneys from *Trf1*^*+/+*^ and *Trf1*^*flox/flox*^ mice. **d** Analysis of the urinary albumin-to-creatinine ratio (UACR), *n* = 6 mice per group. **e** Kaplan–Meier survival curves of *Trf1*^*+/+*^ mice (*n* = 15) and *Trf1*^*flox/flox*^ mice (*n* = 31). **f** Representative images of immunofluorescence (six to eight visual fields) staining for Trf1 (green) and KFP (red) and quantification of the percentage of double KFP^+^TRF1^+^ fibroblasts in *Trf1*^*+/+*^ and *Trf1*^*flox/flox*^ kidneys. **g** Representative images of telomeric immuno-q-FISH in KFP^+^ fibroblasts (Cy3Tel probe (red) and KFP^+^ cells (green)) and quantification of the mean telomere spot intensity in *Trf1*^*+/+*^ and *Trf1*^*flox/flox*^ kidneys. **h** Representative images of telomere-induced foci (TIFs) in 53BP1 (green) and telomeres (red) and quantification of TIFs in KFP^+^ cells in *Trf1*^*+/+*^ and *Trf1*^*flox/flox*^ kidneys. **i** Representative images (15–20 visual fields) and quantification of Masson’s trichrome and Picrosirius red staining in *Trf1*^*+/+*^ and *Trf1*^*flox/flox*^ kidneys. Scale bars, 500 μm. Nuclei are stained with DAPI (blue). The data are presented as the means ± SEMs. An unpaired, two-tailed Student’s *t* test was used. *p < 0.05, **p < 0.01, and ***p < 0.001. The number of mice analyzed per genotype is *n* = 8, if not indicated.

We induced long-term *Trf1* deficiency and subsequent telomere dysfunction in fibroblasts by *i.p*. administering TMX 3 times a week for 8 consecutive weeks and then administering a single booster dose of TMX every week throughout the entire lifespan of the mice to explore the effects of prolonged *Trf1* deletion in renal fibroblasts within kidney tissues in vivo (Fig. 6a). While no discernible changes in body weight or the kidney-to-body weight ratio were observed, significant increases in blood creatinine and BUN levels were observed in *Trf1*^*flox/flox*^ compared with *Trf1*^*+/+*^ mice after 12 and 8 months, respectively (Supplementary Fig. 3c–f). Compared with that in *Trf1*^*+/+*^ mice, the expression of KFP in the interstitium region was noticeably higher, with a significant increase in the ISOM in *Trf1*^*flox/flox*^ mice (Fig. 6b). In conjunction with kidney dysfunction upon long-term TRF1 depletion in fibroblasts, kidneys from *Trf1*^*lox/lox*^ mice appeared pale and damaged in appearance compared with those from *Trf1*^*+/+*^ mice (Fig. 6c). Compared with *Trf1*^*+/+*^ mice, *Trf1*^*flox/flox*^ mice presented an elevated uACR, which coincided with a significant decrease in the survival of *Trf1*^*flox/flox*^ mice after 12 months of TMX administration (Fig. 6d, e). At the HEP, a 13-fold increase in the number of KFP^+^ cells among Col1a2^+^ cells was observed, and the deletion of *Trf1* in fibroblasts was maintained in *Trf1*^*flox/flox*^ mice (Fig. 6f; Supplementary Fig. 3g). No differences in telomere length were observed between *Trf1*^*flox/flox*^ and *Trf1*^*+/+*^ mice, indicating that the deleterious effects of TRF1 abrogation are mediated by telomere uncapping rather than by telomere shortening (Fig. 6g). Indeed, a significant increase in the percentage of KFP^+^pH2aX^+^ double-positive cells were detected in *Trf1*^*flox/flox*^ mice compared with *Trf1*^*+/+*^ mice (Supplementary Fig. 3h). Telomeric immuno-qFISH was performed to ascertain whether this DNA damage was located at telomeres (termed TIFs). *Trf1*^*flox/flox*^ mice presented a 4-fold increase in the number of KFP^+^ cells with more than 2 TIFs (Fig. 6h). Masson’s trichrome and Picosirius red staining revealed signs of both cortical and interstitial fibrosis along with interstitial collagen I and vimentin expression, accompanied by glomerular and tubular injury, as evidenced by the loss of brush borders, cellular vacuolization and atrophy, in the kidneys of *Trf1*^*flox/flox*^ mice (Fig. 6i**;** Supplementary Fig. 4a–c). Persistent *Trf1* deficiency in fibroblasts further aggravated inflammation, hypoxia and microvascular rarefaction in the kidneys (Supplementary Fig. 4d–j). The histopathological analysis at the HEP revealed fibrosis in the lungs, liver and heart, in accordance with the fact that fibroblasts are found in all tissues **(**Supplementary Fig. 5a–c**)**. These pathologies could also contribute to the early mortality of *Trf1*^*flox/flox*^ mice compared with that of *Trf1*^*+/+*^ mice. We performed triple immunostaining for markers of the MMT (F4/80^+^α-SMA^+^KFP^+^), EMT (E-cad^+^α-SMA^+^KFP^+^) and EndMT (CD31^+^α-SMA^+^KFP^+^) to further address whether prolonged *Trf1* deletion resulted in the acquisition of a mesenchymal phenotype via the MMT, EMT and EndMT at the HEP. Notably, in all three transitions, a significant upregulation of KFP^+^SMA^+^ cells was observed, and these cells outnumbered the cells that were in the KFP^+^F4/80^+^SMA^+^ or KFP^+^E-cadherin^+^ SMA^+^ or KFP^+^CD31^+^ SMA^+^ transitions, suggesting that *Trf1*^flox/flox^ renal fibroblasts acquired a mesenchymal phenotype via the MMT, EMT and EndMT over the long term, ultimately leading to fibrosis (Supplementary Fig. 6a–c).

### TRF1 depletion exacerbates folic acid-induced fibrosis

**Fig. 7:**
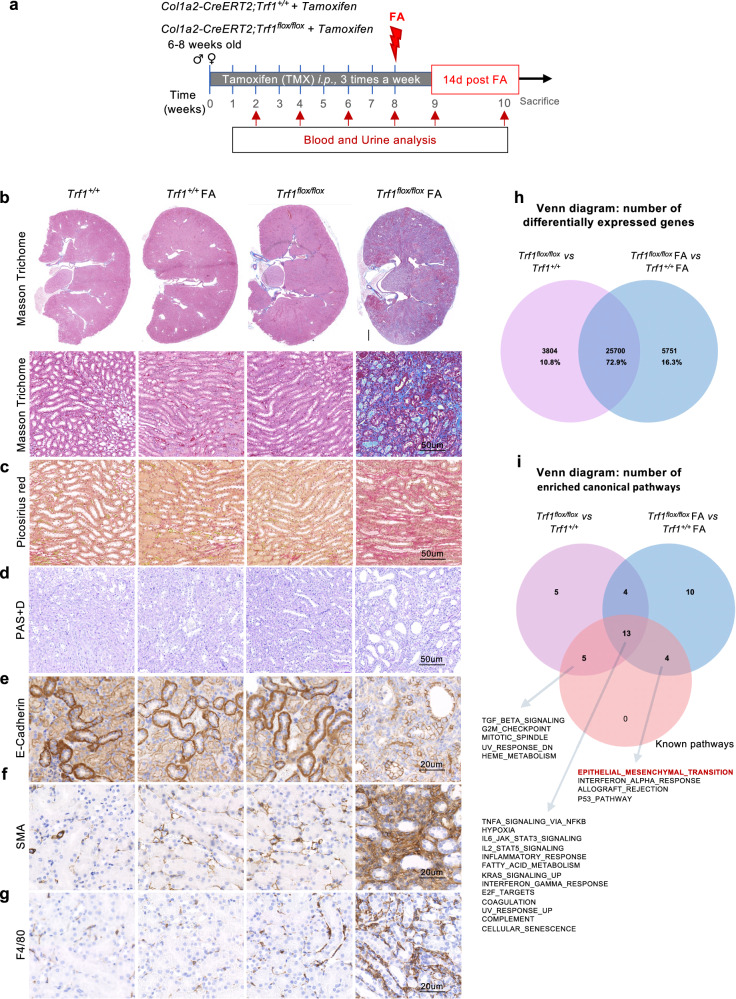
Trf1 depletion exacerbates folic acid-induced fibrosis. **a** Schematic of the experimental approach. A single dose of 125 mg kg^−1^ of body weight FA was administered to *Trf1*^+/+^ and *Trf1*^*flox/flox*^ mice after tamoxifen administration over an 8-week period. Representative images (15–20 visual fields) of (**b**) Masson’s trichrome, (**c**) Picosirius red, (**d**) PAS + D, (**e**) E-cadherin, (**f**) α-SMA, and (**g**) F4/80 staining in the kidneys of *Trf1*^*+/+*^ and *Trf1*^*flox/flox*^ mice that were untreated or treated with FA at a dose of 125 mg kg^−1^. Scale bars, 500 μm. Venn diagram representing the (**h**) number of differentially expressed genes and (**i**) the number of enriched canonical pathways between*Trf1*^*flox/flox*^ and *Trf1*^*+/+*^ mice, *n* = 3 mice in each group; between *Trf1*^*flox/flox*^ FA-treated and *Trf1*^*+/+*^ FA-treated mice, *n* = 4 mice in each group; and known pathways.

We next aimed to address the effects of folic acid (FA) administration after short-term *Trf1* deletion in fibroblasts^24^ (Fig. 7a). Compared with the other groups of mice, the *Trf1*^flox/flox^ FA-treated mice presented increased blood urea nitrogen (BUN) and creatinine levels at 2 days after FA administration (Supplementary Fig. 7a, b). Other blood parameters indicative of kidney dysfunction, including calcium, phosphorus, sodium, albumin, total protein and globulin levels, were also elevated in the *Trf1*^flox/flox^ FA-treated mice (Supplementary Table 2). FA treatment led to increased interstitial fibrosis, including altered tubular morphology, diminished epithelial differentiation, excessive myofibroblast populations, and macrophage infiltration, in *Trf1*^flox/flox^ FA-treated mice compared with those in FA-treated *Trf1*^*+/+*^ and untreated *Trf1*^flox/flox^ and *Trf1*^*+/+*^ mouse cohorts (Fig. 7b–g). We performed RNA sequencing in a set of 3–4 mice from each mouse cohort to further assess the impact of FA treatment on gene expression in TRF1-deficient fibroblasts. We identified 29,504 and 32,637 DEGs when comparing *Trf1*^*flox/lox*^ with *Trf1*^*+/+*^ and FA-treated *Trf1*^*flox/lox*^ with FA-treated *Trf1*^*+/+*^ mice, respectively (GEO IDs: GSE232874) (Fig. 7h). Among these DEGs, 25700 (72.9%) genes were commonly shared between *Trf1*^flox/flox^ and *Trf1*^+/+^ mice and *Trf1*^*flox/flox*^ FA-treated and *Trf1*^*+/+*^ FA-treated mice (Fig. 7h). GSEA revealed that 41% of the observed enriched canonical pathways were coincident in both comparisons, namely, *Trf1*^flox/flox^ vs. *Trf1*^+/+^ mice and *Trf1*^*flox/flox*^ FA-treated vs. *Trf1*^*+/+*^ FA-treated mice (Supplementary Fig. 7c, d). We created a set of pathways that have already been described to be upregulated in kidney fibrosis and compared this pathway set with the pathways that were upregulated in our 2 different groups, namely, *Trf1*^flox/flox^ vs. *Trf1*^+/+^ mice and *Trf1*^*flox/flox*^ FA-treated vs. *Trf1*^*+/+*^ FA-treated mice (Fig. 7i**;** Supplementary Fig. 7e)^32,41,42^. Among these pathways, 13 pathways were commonly shared between *Trf1*^flox/flox^ and *Trf1*^+/+^ mice, *Trf1*^*flox/flox*^ FA-treated and *Trf1*^*+/+*^ FA-treated mice, and known pathways, including TNFa signaling via NFKB, hypoxia, IL6/JAK/STAT3 signaling, IL2/STAT5 signaling and the inflammatory response, among others, in agreement with previous results (Figs. 4, 5g–j, 7i; Supplementary Fig. 2d–f). Notably, the EMT pathway was specifically shared in the comparison between the known pathway set and the upregulated pathway set in *Trf1*^*flox/flox*^ FA-treated vs. *Trf1*^*+/+*^ FA-treated mice and not with the upregulated pathway set in *Trf1*^flox/flox^ vs. *Trf1*^+/+^ mice (Fig. 7i). These findings support the hypothesis that TRF1 depletion over an eight-week period promotes a profibrotic environment and that a low dose of the nephrotoxic agent FA is sufficient to induce full-blown renal fibrosis within the same short-term period, similar to observations made with sustained TRF1 depletion in fibroblasts throughout the mouse lifespan (Fig. 6).

## Discussion

Physiological aging may result in a series of detrimental functional and structural changes in the kidney, including tubular atrophy, loss of nephrons, glomerulonephritis, inflammation, interstitial fibrosis, and chronic kidney disease, which are linked to molecular hallmarks of aging, such as telomere dysfunction and cellular senescence^43–46^. Renal fibrosis is characterized by the infiltration of inflammatory cells, the proliferation and activation of mesenchymal cells, and the progressive deposition of the extracellular matrix (ECM)^5,47,48^. Our previous findings indicated that the global genetic ablation of *Trf1* aggravates kidney fibrosis^14^. However, the specific role of *Trf1* deletion in distinct cell types during the progression of kidney fibrosis remains unexplored. Resident fibroblasts are crucial contributors to kidney fibrosis since they constitute the most important precursor of myofibroblasts^5,6,49^. Renal myofibroblasts are heterogeneous in nature and are believed to originate from various sources, such as the proliferation of resident fibroblasts, differentiation from local fibroblasts or pericytes, direct and complete transition from macrophages via the MMT, and transition from resident endothelial or epithelial cells through the EndMT and EMT^33,34,50–52^.

We aimed to elucidate the role of *Trf1* in kidney fibroblasts, as Trf1 is one of the specific initiators of renal interstitial fibrosis. We studied renal pathological outcomes after the deletion of *Trf1* in kidney-resident fibroblasts over an 8-week period and until the humane end point (HEP). In a short-term study involving the deletion of *Trf1* by administering tamoxifen *i.p*. 3 times a week over a period of 8 weeks, no significant alterations in blood parameters were observed in *Trf1*^*flox/flox*^ mice compared with *Trf1*^*+/+*^ mice (Supplementary Fig. 1d, e), which aligns with observations in human patients, where early stages of kidney disease often go undetected. Short-term depletion of TRF1 led to a substantial increase in the number of interstitial fibroblasts/myofibroblasts, although it was not sufficient to induce fibrosis, suggesting that the time frame may have been insufficient for significant collagen deposition to occur, revealing the initial stages of kidney disease that eventually contribute to fibrosis, and potentially leading to chronic kidney disease (CKD) in the future.

Notably, the increased presence of myofibroblasts or macrophages was predominantly localized to fibroblasts lacking *Trf1*. In response to inflammatory stimulation, macrophages transdifferentiate into myofibroblasts via the MMT, and M1 macrophages are deemed essential for sustaining the proinflammatory state, contributing to progressive renal injury^33,34^. Consistent with these findings, our observations indicate that approximately one-third of *Trf1*-deficient fibroblasts undergo the MMT, primarily involving M1 macrophages, during short-term *Trf1* deletion. Conversely, the uncontrolled polarization of M2 macrophages was noted at the HEP, leading to fibrotic remodeling of organs^53^.

The Tgf-β pathway serves as a pivotal stimulator for fibroblasts, which are master regulators of renal inflammation and tubulointerstitial fibrosis^37,54,55^. Elevated expression of the *Tgf-β1* mRNA and protein is evident in patients with fibrotic kidney diseases, including IgA nephropathy, focal and segmental glomerulonephritis, lupus nephritis, and diabetes^55,56^. Tgf-β is a crucial regulator of the EMT that orchestrates numerous pathological processes associated with CKD^3,39,57–59^. One of the hallmarks of the EMT is the loss of epithelial surface markers and the acquisition of mesenchymal markers^37,39,60,61^. During the EMT, the downregulation of E-cadherin can be mediated by its transcriptional repression through the binding of EMT transcription factors (EMT-TFs). As anticipated, kidneys lacking *Trf1* in fibroblasts presented upregulated Tgf-β signaling, increased expression of EMT transcription factors and decreased E-cadherin expression, suggesting that fibroblasts lacking TRF1 activate the EMT.

Inflammation is considered a key driver of kidney fibrosis^43^ and induces the production of proinflammatory cytokines, such as TNFα, to activate NF-κB signaling. TNF-α is a potent cytokine that mediates inflammatory tissue damage in the kidney and specifically activates the proinflammatory cytokines IL-1β and IL-6^62,63^. Systemic inflammation in end-stage renal disease is widely recognized as a substantial risk factor leading to increased mortality in affected individuals^19^ NF-*κ*B is considered to promote inflammation during renal disease, with evidence correlating NF-*κ*B activation in the kidneys of patients with glomerulonephritis, diabetic nephropathy, and AKI^62,64^ and animal models of renal inflammation and injury^65^. The JAK/STAT signaling pathway, which is crucial for immune and inflammatory responses, is also implicated in renal diseases^26,66,67^. In the present study, the gene expression levels of inflammatory cytokines and components of the immune response, including IL-1β, TNFα and IL-6, were increased in kidneys lacking *Trf1* in fibroblasts.

Peritubular capillary rarefaction is a hallmark of CKD and of the acute kidney injury-to-CKD transition^68,69^. We observed downregulation of the angiogenesis pathway with the loss of CD31/PECAM^+^ and VEGFR2^+^ endothelial cells and increased vascular permeability and hypoxia in kidneys lacking TRF1 in fibroblasts. Multiple studies have identified the EndMT in both experimental models of kidney fibrosis and biopsies from CKD patients^69–72^. We showed that *Trf1*-deficient cells undergo the EndMT by immunostaining with CD31, α-SMA, and Katushka. Indeed, one-third of Katushka-positive cells were also positive for both CD31 and α-SMA, suggesting that the deletion of *Trf1* in fibroblasts activated the EndMT process.

We extended our investigation to examine the consequences of sustained *Trf1* abrogation in fibroblasts throughout the lifespan of mice, exploring the potential correlation with the development of chronic fibrosis in the kidneys. Notably, persistent DNA damage at telomeres in kidney fibroblasts resulted in kidney failure, as evidenced by an increase in the urinary albumin-to-creatinine ratio (uACR) and a decrease in mouse survival, which coincided with the onset of kidney interstitial fibrosis. Furthermore, our findings indicated that the processes of the MMT, partial EMT and EndMT were augmented in a time-dependent manner with prolonged *Trf1* deletion, paralleling the progression of fibrosis. In the short term, we observed a greater than 10-fold increase in the number of *Trf1*-deleted cells undergoing the MMT (KFP^+^F4/80^+^α-SMA^+^), EMT (KFP^+^E-cad^+^α-SMA^+^) and EndMT (KFP^+^CD31^+^α-SMA^+^) compared with *Trf1*^*+/+*^ cells. Interestingly, at the HEP, the percentage of *Trf1*-deleted cells undergoing the MMT, EMT and EndMT was greatly reduced compared to that in the control group. The predominant cell population (90%) observed consisted of activated myofibroblasts (α-SMA^+^KFP^+^), suggesting that the transitions initiated in the short term ultimately led to excessive deposition of the extracellular matrix (ECM), a definitive hallmark of the kidney fibrosis phenotype, and exacerbated the classical features observed in patients with CKD.

In conclusion, we have demonstrated for the first time that telomere dysfunction in renal fibroblasts activates an inflammatory cascade, vascular rarefaction, and G2 arrest, causing fibroblasts to undergo various transitions (MMT, partial EMT, and EndMT) in the context of acute kidney injury (AKI) and maladaptive repair, exacerbating the classical features observed in patients. This persistent injury leads to the excessive accumulation of collagen and ECM components, resulting in kidney fibrosis, as observed in patients with CKD, and reduced dynamic compliance. Despite the increasing number of patients with CKD and kidney fibrosis and the development of therapeutic agents ranging from chemical compounds to gene therapies for renal fibrosis, clinical translation from the bench to the bedside is often limited due to the slow progression of the disease, the heterogeneity of patients, and the lack of noninvasive biomarkers for renal fibrosis^73^. Thus, the early detection and accurate assessment of the cell types responsible for kidney fibrosis are crucial for the application of timely interventions as a primary measure and the discovery of new cell-targeted therapies to treat chronic kidney disease (CKD). Several studies have shown a correlation between a short telomere length and the onset of CKD^46,74–76^ Rationally designed targeted gene therapies might exhibit increased specificity, bioavailability, and inhibitory mechanisms that have more significant effects on clinical outcomes than the small molecules that are currently available^77^. Our study highlights the human characteristics of CKD development starting from a condition of kidney disease. This study broadens our understanding of key cellular and molecular mechanisms and provides insights into the genetic mechanisms underlying kidney disease mediated by telomere dysfunction in resident fibroblasts.

## Supplementary information

Supplementary information

## Data Availability

The authors declare that the data supporting the findings of this study are available within the paper (and its Supplementary Information files). Source data are provided with this paper.
