# Author Correction: Renal fibroblasts are involved in fibrogenic changes in kidney fibrosis associated with dysfunctional telomeres

**DOI:** 10.1038/s12276-024-01370-4

**Published:** 2024-12-04

**Authors:** Sarita Saraswati, Paula Martínez, Rosa Serrano, Diego Mejías, Osvaldo Graña-Castro, Ruth Álvarez Díaz, Maria A. Blasco

**Affiliations:** 1 Telomeres and Telomerase Group–Fundacion Humanismo y Ciencia, Molecular Oncology Program, Spanish National Cancer Centre (CNIO), Melchor Fernández Almagro 3, E-28029 Madrid, Spain; 2 Confocal Microscopy Unit, Biotechnology Program, Spanish National Cancer Centre (CNIO), Melchor Fernández Almagro 3, E-28029 Madrid, Spain; 3 Advanced Optical Microscopy Unit, UCCTs, Instituto de Salud Carlos III (ISCIII), E-28220 Majadahonda, Madrid Spain; 4 Bioinformatics Unit, Structural Biology and Biocomputing Program, Spanish National Cancer Centre (CNIO), Melchor Fernández Almagro 3, E-28029 Madrid, Spain; 5 Department of Basic Medical Sciences, Institute of Applied Molecular Medicine (IMMA-Nemesio Díez), School of Medicine, San Pablo-CEU University, CEU Universities, Boadilla del Monte, Madrid, Spain

**Keywords:** Mechanisms of disease, Gene expression

Correction to: *Experimental & Molecular Medicine* 10.1038/s12276-024-01318-8, published online 01 October 2024

After online publication of this article, the authors noticed an error in the acknowledgment section.

The correct statement of this article should have read as below.

Research in the Blasco laboratory is funded by the Spanish State Research Agency, Ministry of Science and Innovation, and cofunded by the European Regional Development Fund (AF2017-82623-R and SAF2015-72455-EXP), the Comunidad de Madrid Project (S2017/BMD-3770), the World Cancer Research Project (16-1177), the European Research Council (SHELTERINS GA882385), Fundación Botín (Spain) and philanthropic support from Fundación Humanismo y Ciencia.

The authors apologize for any inconvenience caused.

The original article has been corrected.

