# Supplementary material for: Renal fibroblasts are involved in fibrogenic changes in kidney fibrosis associated with dysfunctional telomeres
Source: Exp Mol Med. 2024 Oct 1;56(10):2216–30. doi: 10.1038/s12276-024-01318-8 (PMC11541748; doi:10.1038/s12276-024-01318-8)
Supplement: Supplementary file 1 — Supplementary information [file 12276_2024_1318_MOESM1_ESM.pdf]

Supplementary Fig. 1

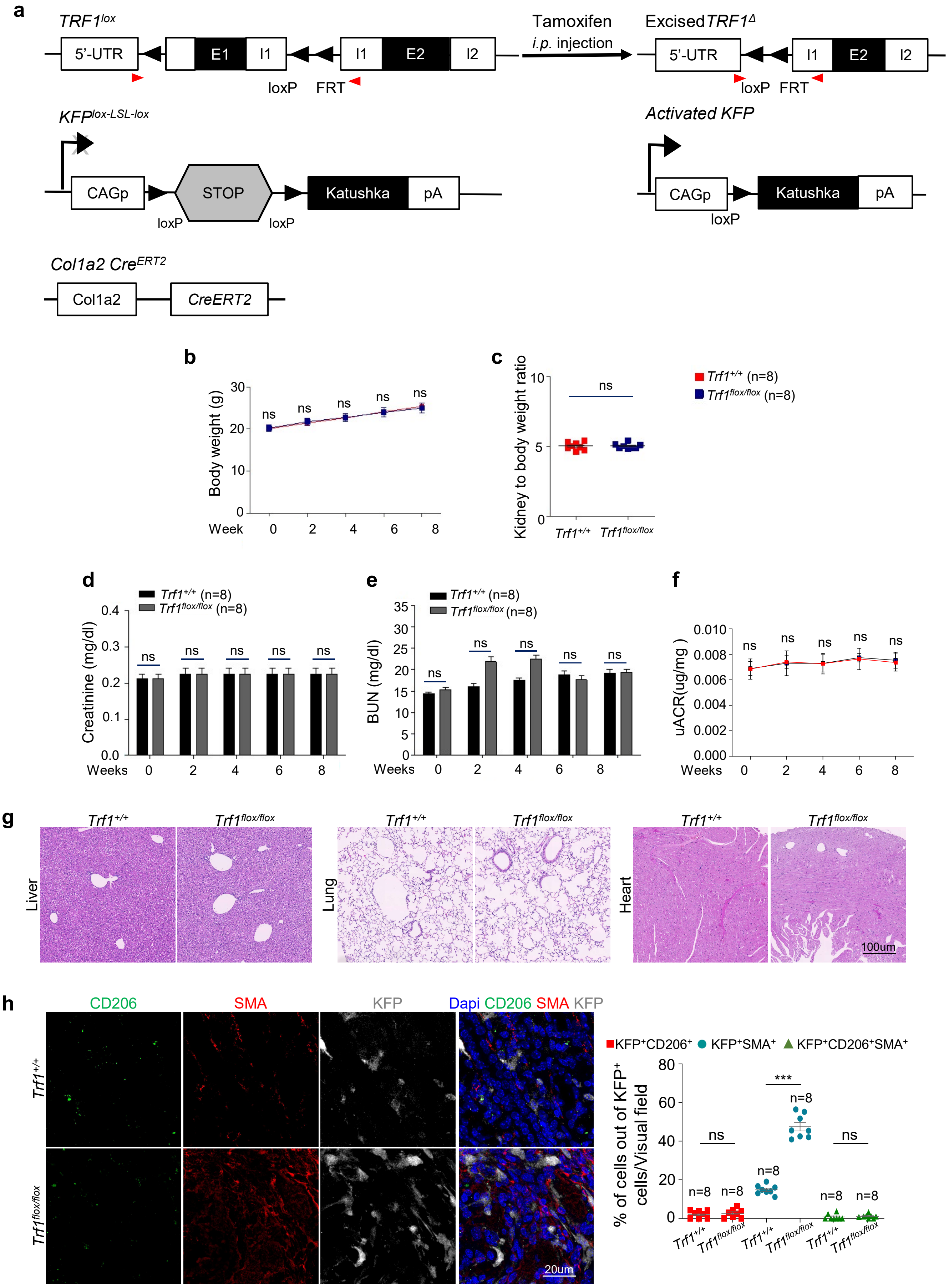

Supplementary Fig. 2

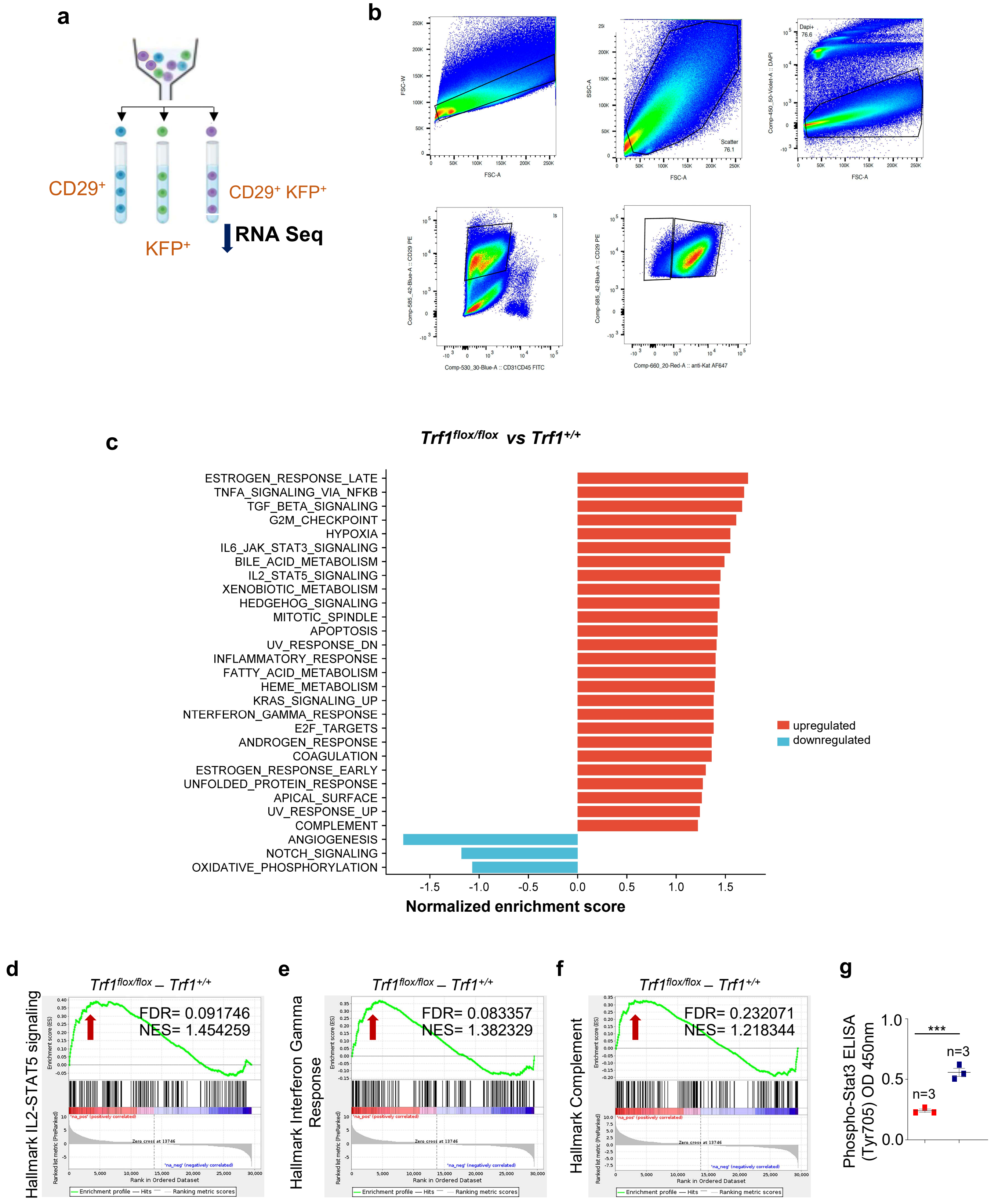

Supplementary Fig. 3

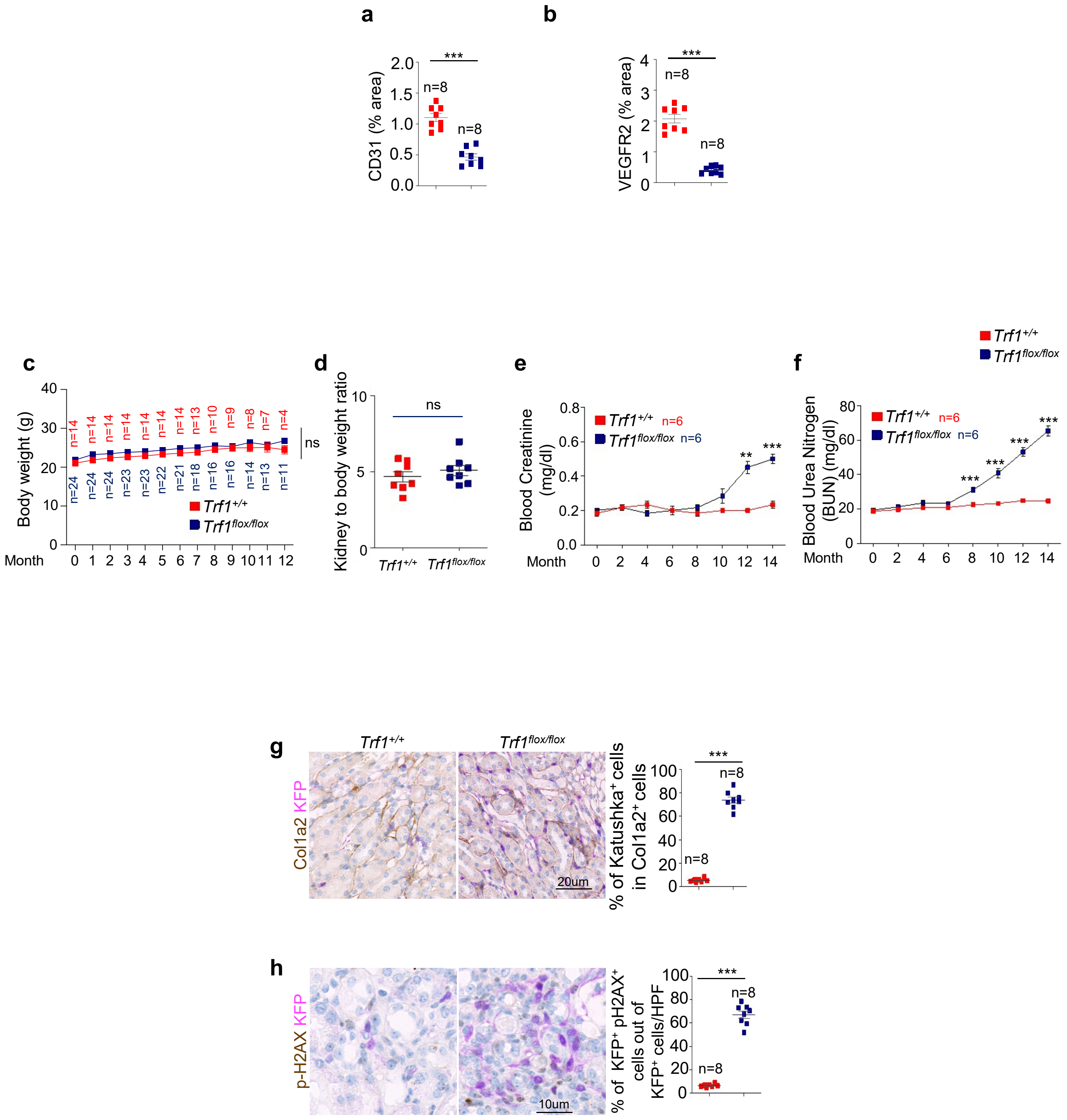

Supplementary Fig. 4

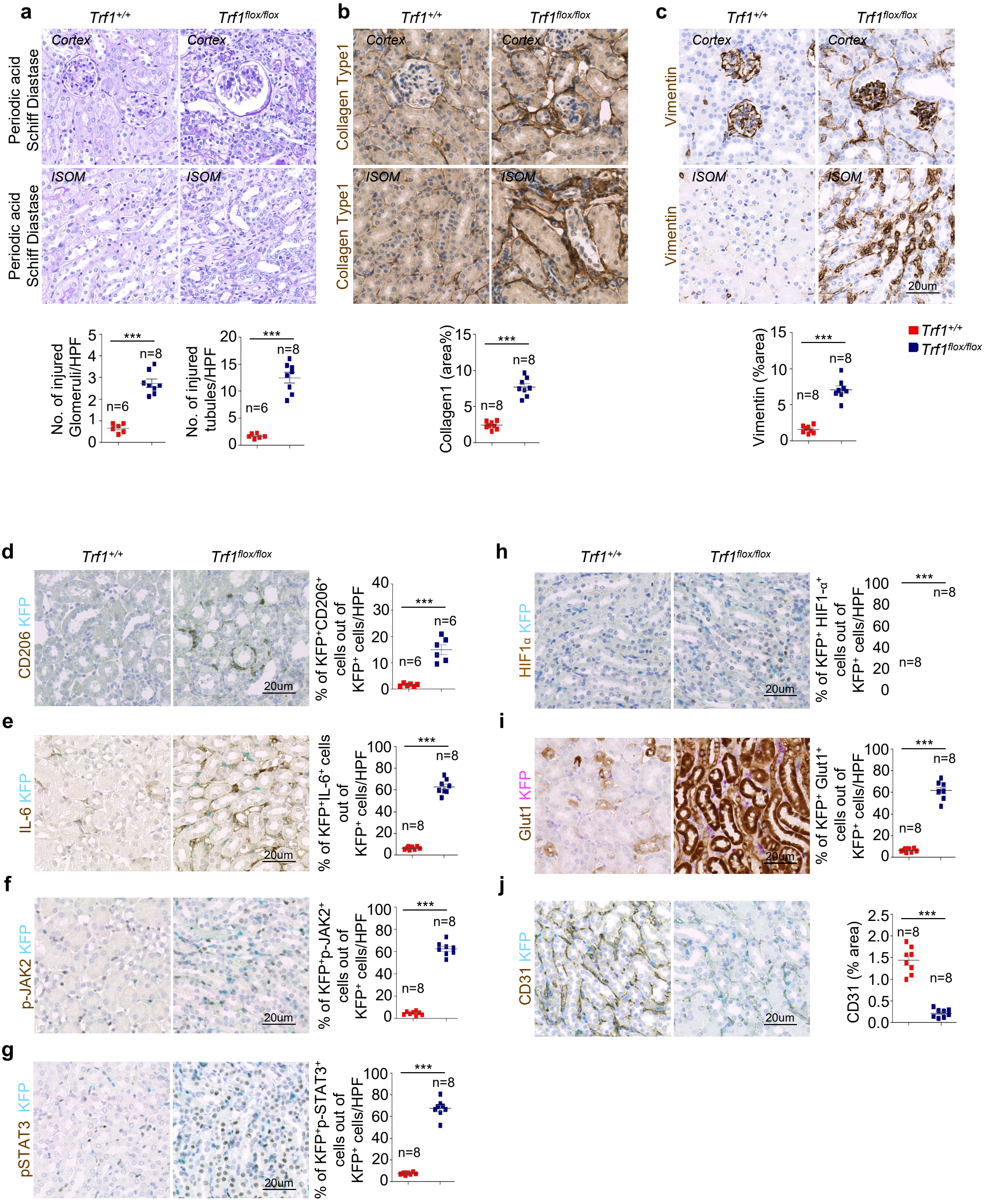

Supplementary Fig. 5

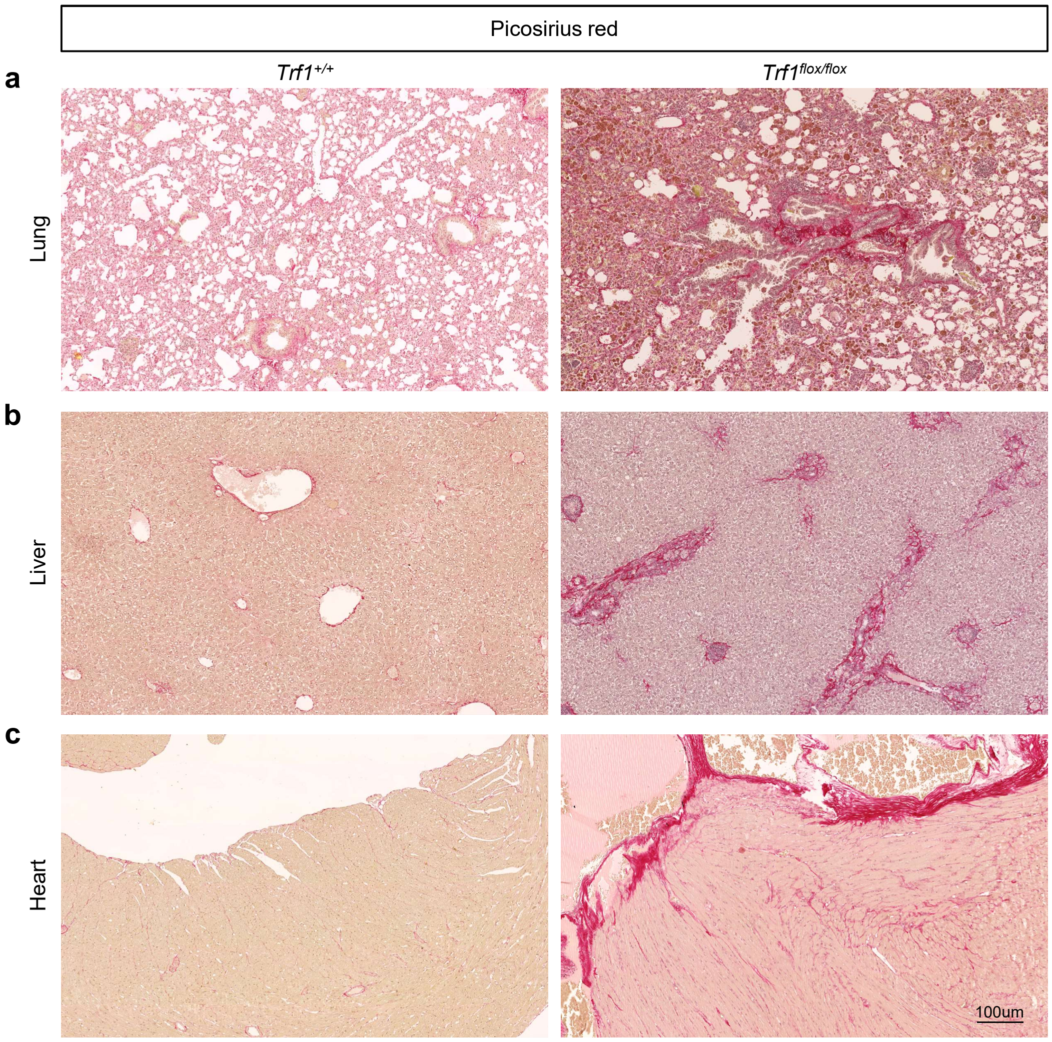

Supplementary Fig. 6

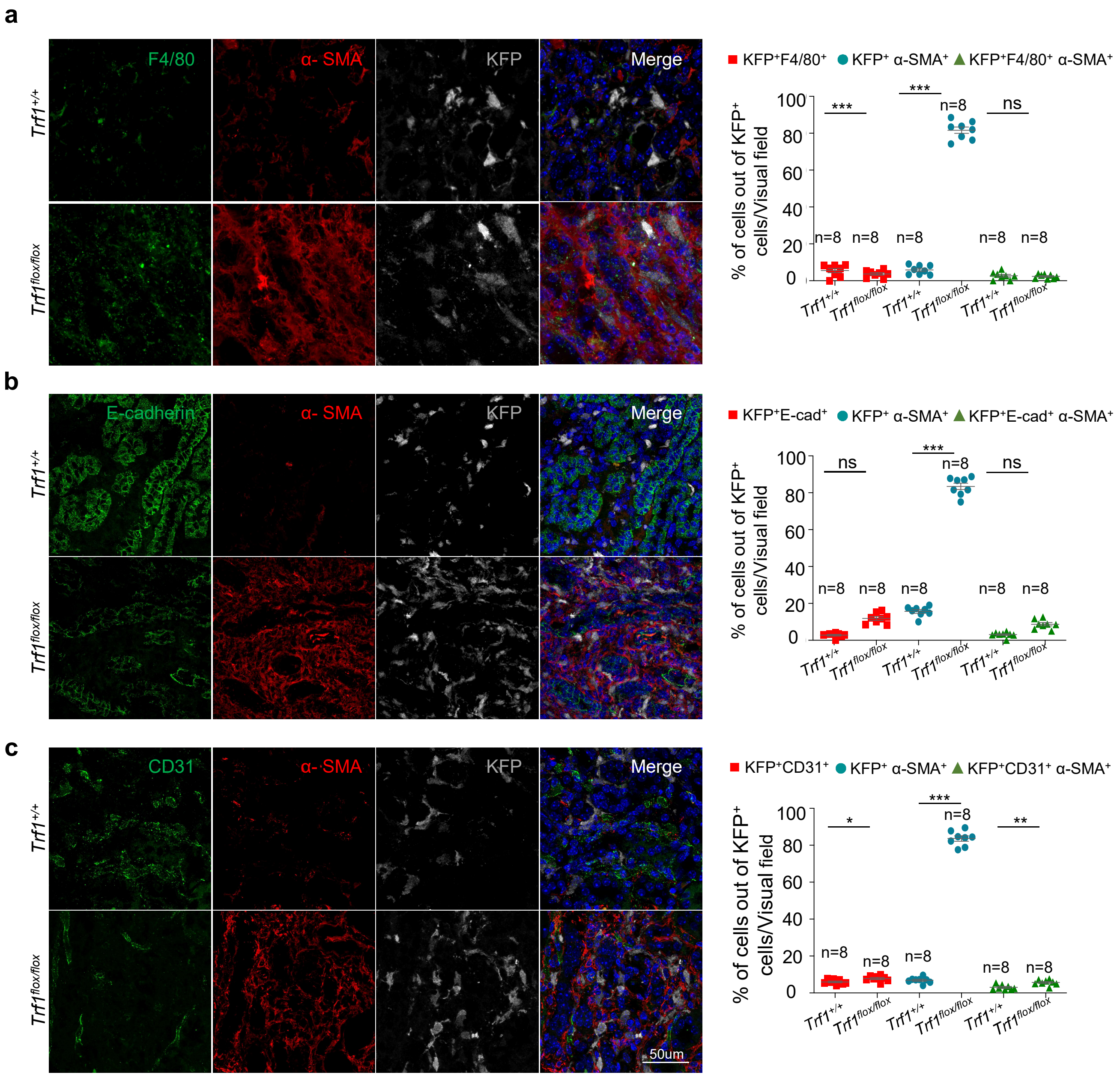

Supplementary Fig. 7

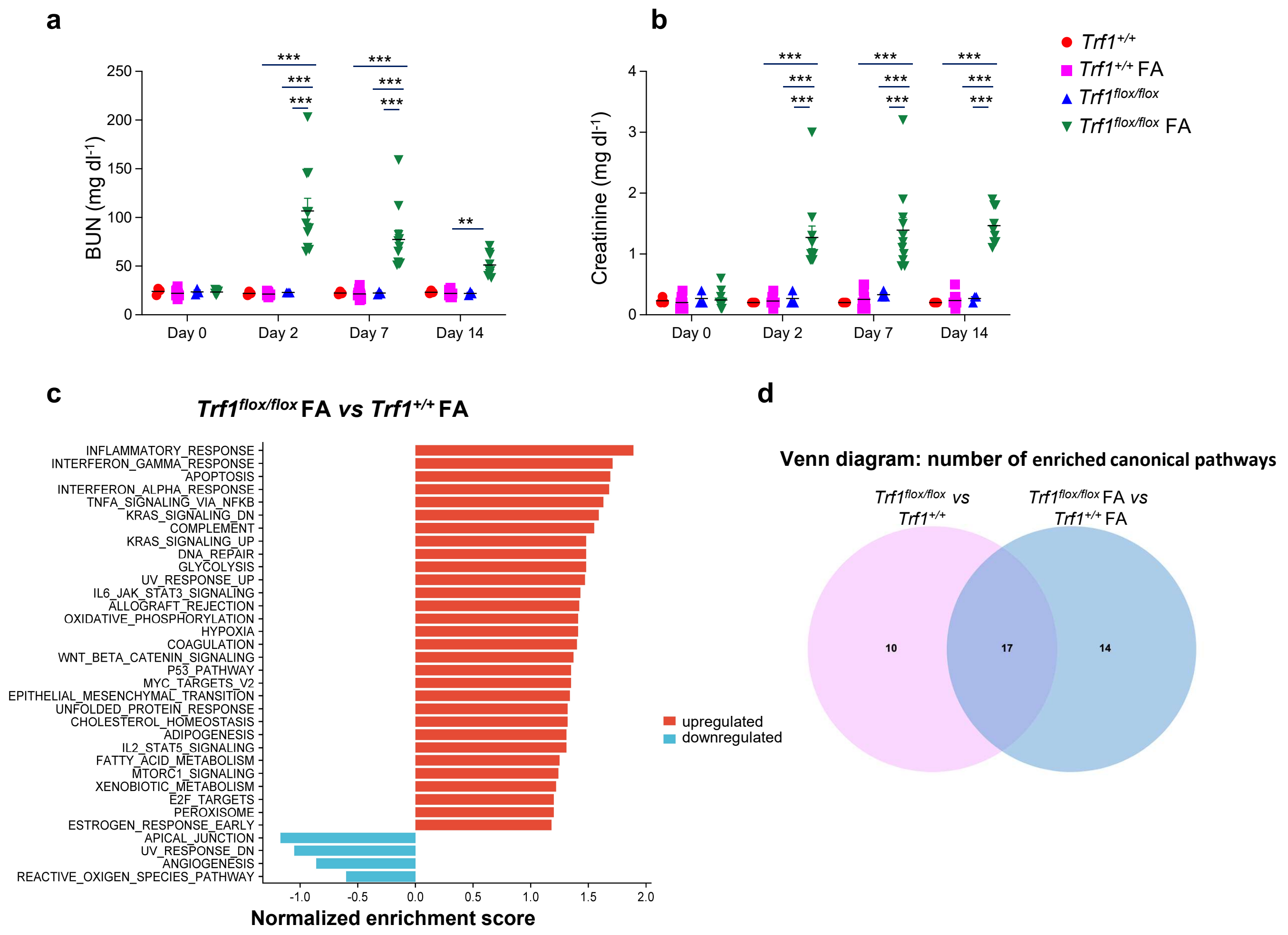

e

KNOWN PATHWAYS UPREGULATED IN KIDNEY FIBROSIS

- HALLMARK_ALLOGRAFT_REJECTION
- HALLMARK_INTERFERON_GAMMA_RESPONSE
- HALLMARK_INFLAMMATORY_RESPONSE
- HALLMARK_TNFA_SIGNALING_VIA_NFKB
- HALLMARK_INTERFERON_ALPHA_RESPONSE
- HALLMARK_IL6_JAK_STAT3_SIGNALING
- HALLMARK_EPITHELIAL_MESENCHYMAL_TRANSITION
- HALLMARK_COMPLEMENT
- HALLMARK_KRAS_SIGNALING_UP
- HALLMARK_IL2_STAT5_SIGNALING
- HALLMARK_E2F_TARGETS
- HALLMARK_G2M_CHECKPOINT
- HALLMARK_MITOTIC_SPINDLE
- HALLMARK_UV_RESPONSE_DN
- HALLMARK_COAGULATION
- HALLMARK_HYPOXIA
- HALLMARK_TGF_BETA_SIGNALING
- HALLMARK_P53_PATHWAY
- HALLMARK_HEME_METABOLISM
- HALLMARK_FATTY_ACID_METABOLISM
- HALLMARK_UV_RESPONSE_UP
- HALLMARK_ CELLULAR_SENESCENCE

Supplementary Information

Supplementary Figures

Supplementary Fig. 1. Conditional deletion of *Trf1* in fibroblasts

a Generation of the conditional knockout mouse model in which *Trf1* was deleted in fibroblasts using the Cre recombinase driven by the *Col1a2* promoter. **b** Body weight and **c** kidney to body weight ratio in $Trf1^{flox/flox}$ compared to $Trf1^{+/+}$ mice. **d** **b**lood creatinine **e** blood urea nitrogen (BUN) **f** urinary albumin-to-creatinine ratio (uACR) in $Trf1^{flox/flox}$ compared to $Trf1^{+/+}$ mice. **g** Representative H & E of liver, lung and heart in $Trf1^{flox/flox}$ compared to $Trf1^{+/+}$ mice. **h** Representative images (six to eight visual fields) and quantifications of immunolabeling for CD206, α-SMA and KFP in $Trf1^{flox/flox}$ compared to $Trf1^{+/+}$ mice. Nuclei are stained with DAPI (blue). Data are represented as mean ± SEM. An unpaired, two-tailed Student's *t*-test was used. **p*< 0.05, ***p* < 0.01, ****p* < 0.001. The number of mice analyzed per genotype is n=8, if not indicated.

Supplementary Fig. 2. Flow cytometry cell isolation

a Schematic summary of the experimental protocol used to isolate $CD29^{+}$ or $CD29^{+}$ KFP^{+} or or KFP^{+} cells from $Trf1^{flox/flox}$ and $Trf1^{+/+}$ kidneys. **b** Gating strategy for isolating fibroblasts from $Trf1^{flox/flox}$ and $Trf1^{+/+}$ kidneys. **c** Results of GSEA Hallmark pathways (FDR < 25%) in $Trf1^{flox/flox}$ compared to $Trf1^{+/+}$ mice**.** GSEA enrichment plots of hallmark gene dataset showed the enrichment in **d** IL2-STAT5 signaling **e** Interferon Gamma Response **f** Complement system in the kidneys of $Trf1^{+/+}$ and $Trf1^{flox/flox}$ mice. **g** ELISA of pSTAT3 in the kidneys of $Trf1^{+/+}$ and $Trf1^{flox/flox}$ mice. All groups, *n* = 3 mice for each group. Data are represented as mean ± SEM. An unpaired, two-tailed Student's *t*-test was used. **p*< 0.05, ***p* < 0.01, ****p* < 0.001.

Supplementary Fig. 3. Effects of short term and long-term fibroblast-specific deletion of *Trf1*

Representative immunostainings (15-20 visual fields) and quantifications **a** $CD31^+$ **b** $VEGFR2^+$in $Trf1^{+/+}$ and $Trf1^{flox/flox}$ kidneys **c** Body weight **d** kidney to body weight ratio **e** Blood creatinine **f** blood urea nitrogen (BUN) in $Trf1^{flox/flox}$ compared to $Trf1^{+/+}$ mice at HEP. Representative immunostainings (15-20 visual fields) and quantifications **g** $Col1a2^+KFP^+$ **h** p-$H2AX^+KFP^+$ in $Trf1^{+/+}$ and $Trf1^{flox/flox}$ kidneys**.** Data are represented as mean ± SEM. An unpaired, two-tailed Student's *t*-test was used. $^*p< 0.05$, $^{**}p < 0.01$, $^{***}p < 0.001$. The number of mice analyzed per genotype is n=8, if not indicated.

Supplementary Fig. 4. Fibroblast-specific deletion of *Trf1* exacerbates inflammatory response, hypoxia, and vascular rarefaction at HEP

Representative images (15-20 visual fields) and quantifications of **a** PAS+D **b** Collagen I **c** Vimentin **d** $CD206^+$ KFP^+ **e** $IL6^+$ KFP^+ **f** p-$JAK2^+$ KFP^+ **g** p-$STAT3^+$ KFP^+ **h** HIF-1α$^+$ KFP^+ **i** $Glut1^+$ KFP^+ **j** CD31 in $Trf1^{+/+}$ and $Trf1^{flox/flox}$ kidneys. Data are represented as mean ± SEM. An unpaired, two-tailed Student's *t*-test was used. $^*p< 0.05$, $^{**}p < 0.01$, $^{***}p < 0.001$. The number of mice analyzed per genotype is n=8, if not indicated.

Supplementary Fig. 5. Long-term and persistent fibroblast-specific deletion of *Trf1* leads to signs of fibrosis in different organs at HEP

Representative images and quantifications of Picosirius red stainings in **a** Lung **b** Liver **c** Heart in $Trf1^{+/+}$ and $Trf1^{flox/flox}$ mice. Scale bars,100μm**.** The number of mice analyzed per genotype is n=8, if not indicated.

Supplementary Fig. 6. Persistent long-term fibroblast-specific deletion of *Trf1* leads to acquisition of a mesenchymal phenotype via MMT, partial EMT and EndMT at HEP

a Representative image (six to eight visual fields) and quantifications of immunolabeling for F4/80, α-SMA and KFP in $Trf1^{+/+}$ and $Trf1^{flox/flox}$ kidneys. **b** Representative images (six to eight visual fields) and quantifications of immunolabeling for E-cadherin, α-SMA and KFP in $Trf1^{+/+}$ and $Trf1^{flox/flox}$ kidneys. **c** Representative images (six to eight visual fields) and quantifications

of immunolabeling for CD31, α-SMA and KFP in *Trf1*$^{+/+}$ and *Trf1*$^{flox/flox}$ kidneys. Nuclei are stained with DAPI (blue). Data are represented as mean ± SEM. An unpaired, two-tailed Student's *t*-test was used. *$p < 0.05$, **$p < 0.01$, ***$p < 0.001$. The number of mice analyzed per genotype is n=8, if not indicated.

Supplementary Fig. 7. Effect of Trf1 deletion on Folic acid-induced fibrosis model.

a Blood urea nitrogen (BUN) **b** blood creatinine in the kidneys of *Trf1*$^{+/+}$ and *Trf1*$^{flox/flox}$ mice untreated or treated with FA at a dose of 125 mg kg^{-1}. Representative images (10 to 15 visual fields) **c** Results of GSEA Hallmark pathways (FDR < 25%) **d** Venn diagram representing number of enriched canonical pathways in the kidneys of *Trf1*$^{+/+}$ and *Trf1*$^{flox/flox}$ mice untreated or treated with FA at a dose of 125 mg kg^{-1}. **e** Known pathways set. Data are represented as mean ± SEM. An unpaired, two-tailed Student's *t*-test was used. *$p < 0.05$, **$p < 0.01$, ***$p < 0.001$. The number of mice analyzed per genotype is n=8, if not indicated.

Supplementary Table 1: RT–qPCR **primers**

**Gene**	**Sequence (5'- 3')**
Acta2 F	ATCACCAACTGGGACGACAT
Acta2 R	AGTGTCGGATGCTCTTCAGG
Ca9 F	TACGAACAGTTGCTGTCCCA
Ca9 R	GGTAGTAGCGGCTGAGGTCC
Cd4 F	ACCAGAGGAAGATTCTGGGG
Cd4 R	TCAAAACGATCAAACTGCGA
Cd8a F	GATTGGACTTCGCCTGTGAT
Cd8a R	GCCTTCCTGTCTGACTAGCG
Cd8b F	ATGTGAAGCCAGAGGACAGTG
Cd8b R	GGGGCAGTTGTAGGAAGGAC
Cdh1 F	*GCAGAACTGTCCCTGTCC CAG*
Cdh1 R	*GAACAGCACGTACACAGC CCT*
Cdkn1a F	TGTCCGTCAGAACCCATGC
Cdkn1a R	AAAGTCGAAGTTCCATCGCTC
Cdkn1b F	AACGTGCGAGTGTCTAACGG
Cdkn1b R	CCCTCTAGGGGTTTGTGATTCT
Cxcl10 F	AGGGTCGTGACTGAAAACCA
Cxcl10 R	AGCGTGGTGACCATTTCAA
Emr1 F	GCACCATCTTGCTGGAGACT
Emr1 R	TTCATTGATGACTTTGCTTTCG
GAPDH F	AGGTCGGTGTGAACGGATTTG
GAPDH R	TGTAGACCATGTAGTTGAGGTCA
Hif1*α* F	TAGTAAGCGGAGATCGGGTG
Hif1*α* R	CTGAGGGGTTATGGATGCAA

IL15rα- F	TTAAGCGGAAAGCTGGAACA
IL15rα R	GTCATTTTGGAACTGTGGGG
IL17rb F	GTGTGGCAGCAGAGGAGTTT
IL17rb R	TGTTGTTTTTGCCACTCACG
IL18r1 F	TAGTCTCACTTGCTGGGCG
IL18r1 R	GATGCATGTTTAGGCTTCCACT
IL1β F	GCTGAAAGCTCTCCACCTCA
IL1β R	CCAAGGCCACAGGTATTTTG
IL1r1 F	CTCATTTGTCTCATGGTGCCT
IL1r1 R	TGGAGTAAGAGGACACTTGCG
IL6st F	TTGATTGCAAACGTGGATTG
IL6st R	TGAATCGGAAACAAGTTCGC
Kdr F	GCGACCCCAAATTCCATTAT
Kdr R	CCGGCTCTTTCGCTTACTG
Lnc2 F	CAATGTCACCTCCATCCTGG
Lnc2 R	CCTGGAGCTTGGAACAAATG
Nfkβ1 F	TCCGGGAGCCTCTAGTGAG
Nfkβ1 R	CTTTGCAGGCCCCACATAGT
Nfkβ2 F	GGCCGGAAGACCTATCCTACT
Nfkβ2 R	CTACAGACACAGCGCACACT
Pecam F	AAACCGTATCTCCAAAGCCAG
Pecam R	ACAGGTCCAACAACTCCCCT
Smad3 F	TGAACACCAAGTGCATTACCA
Smad3 R	AGGCGGCAGTAGATAACGTG
Snai1 F	ACCCTGCTGGTATCTCTCCC
Snai1 R	TCTTCACATCCGAGTGGGTT
Snai2 F	GGCTGCTTCAAGGACACATT

Snai2 R	GTGCCCTCAGGTTTGATCTG
Terf1 F	TGGAAAATGAGAAAGCGAGG
Terf1 R	TTTCTGTAGACTGCTGGCCATT
Tgfb1 F	GGAGAGCCCTGGATACCAAC
Tgfb1 R	ATCCACTTCCAACCCAGGTC
Tnfaip3 F	AGAAGTGTCCAGGCTTCCCT
Tnfaip3 R	ATGCATGAGGCAGTTTCCAT
Traf1 F	ATGCAGAGCAGACAACCTCC
Traf1 R	TGAAGGAACAGCCAACACCT
Twist F	CACGCTGCCCTCGGACAA
Twist R	GGGACGCGGACATGGACC
Zeb1 F	CCAAGAACTGCTGGCAAGA
Zeb1 R	TTCGGATCATGGTTTTGCTC
Zeb2 F	AAACAAGCCAATCCCAGGAG
Zeb2 R	GAGGGTTTGCAAGGCTATCA

Supplementary Table 2: Blood parameters of mice at day 0 2, 7 and 14 post-FA.

**Parameters**		**Trf1$^{+/+}$**	**Trf1$^{+/+}$ FA**	***Trf1$^{flox/flox}$***	***Trf1$^{flox/flox}$* FA**
Calcium(mg/dL)	Day 0	10.3 ± 0.1	10.32 ± 0.200	10.43 ± 0.305	10.35 ± 0.206
	Day 2	10.06 ± 0.057	10.23 ± 0.170	10.3 ± 0.173	11.748 ± 0.787^{a,b,c}
	Day 7	10.4 ± 0.173	10.5 ± 0.260	10.46 ± 0.305	12.25 ± 0.738 ^{a,b,c}
	Day 14	10.36 ± 0.115	10.46 ± 0.224	10.23 ± 1.527	12.64 ± 0.320 ^{a,b,c}
Phosphorus (mg/dL)	Day 0	5.96 ± 0.208	5.85 ± 1.073	6.03 ± 0.378	5.85.87 ± 0.452
	Day 2	5.6 ± 0.529	5.32 ± 0.827	5.3 ± 0.953	8.12 ± 1.920 ^{b,c,d}
	Day 7	5.9 ± 0.264	5.66 ± 0.812	5.96 ± 0.776	9.44 ± 1.999 ^{a,b,c}
	Day 14	6.33 ± 0.321	6.23 ± 0.320	6.43 ± 0.208	10.17 ± 1.992 ^{a,b,c}
Sodium (mMol/L)	Day 0	147 ± 1.732	145.45 ± 2.018	144.66 ± 1.527	145.09 ± 1.578
	Day 2	145 ± 3	143.5 ± 3.439	145.66 ± 0.577	146.54 ± 2.207 ^{a,b,f}
	Day 7	143.33 ± 4.041	141.18 ± 2.60	144.33 ± 2.081	147.09 ± 2.913 ^{a,b}
	Day 14	143.66 ± 0.577	140 ± 2.449	143 ± 3.605	149.77 ± 2.728 ^{a,b,c,g,h}
Albumin (g/dL)	Day 0	4.26 ± 0.115	4.24 ± 0.191	3.76 ± 1.265	4.17 ± 0.110
	Day 2	3.73 ± 0.208	3.8 ± 0.2	3.55 ± 1.208	3.53 ± 0.374
	Day 7	3.73± 0.057	3.97 ± 0.261	3.45 ± 1.140	3.72 ± 0.228 ^{a,b,f}
	Day 14	3.76± 0.152	3.99 ± 0.273	3.52 ± 1.159	4.26 ± 0.187 ^{a,b,c}
Total Protein (g/dL)	Day 0	5.73 ± 0.152	5.62 ± 0.148	5.66 ± 0.152	5.52 ± 0.100
	Day 2	5.5 ± 0.1	5.5 ± 0.189	5.66 ± 0.251	6.07 ± 0.462 ^{e,f}
	Day 7	5.46 ± 0.208	5.56 ± 0.287	5.66 ± 0.057	6.50 ± 0.717 ^{a,b,g}
	Day 14	5.56 ± 0.404	5.62 ± 0.319	5.6 ± 0.2	6.85 ± 0.461 ^{a,b,c}
Globulin(g/dL)	Day 0	1.46 ± 0.057	1.43 ± 0.220	1.43 ± 0.057	1.40 ± 0.943
	Day 2	1.56 ± 0.057	1.609 ± 0.157	1.46 ± 0.152	2.26 ± 0.576 ^{a,b,c}
	Day 7	1.73 ± 0.057	1.67 ± 0.110	1.66 ± 0.115	2.62 ± 0.431 ^{a,b,c}
	Day 14	1.56 ± 0.110	1.64 ± 0.150	1.6 ± 0.3	2.83 ± 0.269 ^{a,b,c}

$^{a}p < 0.001$ *Trf1$^{+/+}$ vs. Trf1$^{flox/flox}$* FA*;* $^{b}p < 0.001$ *Trf1$^{+/+}$FA vs Trf1$^{flox/flox}$* FA; $^{c}p < 0.001$ *Trf1$^{flox/flox}$ vs. Trf1$^{flox/flox}$ FA*

$^{d}p < 0.01$ *Trf1$^{+/+}$ vs. Trf1$^{flox/flox}$* FA; $^{e}p < 0.001$ *Trf1$^{flox/flox}$ vs. Trf1$^{flox/flox}$ FA*

$^{f}p < 0.05$ *Trf1$^{flox/flox}$ FA vs. Trf1$^{+/+}$ FA;* $^{g}p < 0.01$ *Trf1$^{flox/flox}$ vs. Trf1$^{flox/flox}$ FA;* $^{h}p < 0.01$ *Trf1$^{+/+}$ vs. Trf1$^{flox/flox}$ FA*

Data are presented as mean ± standard deviation of the mean.
